# Integration of genomics, metagenomics, and metabolomics to identify interplay between susceptibility alleles and microbiota in adenoma initiation

**DOI:** 10.1186/s12885-020-07007-9

**Published:** 2020-06-29

**Authors:** Jacob E. Moskowitz, Anthony G. Doran, Zhentian Lei, Susheel B. Busi, Marcia L. Hart, Craig L. Franklin, Lloyd W. Sumner, Thomas M. Keane, James M. Amos-Landgraf

**Affiliations:** 1 Department of Veterinary Pathobiology, University of Missouri, Columbia, MO 65201 USA; 2 Present Address: F. Widjaja Foundation Inflammatory Bowel and Immunobiology Research Institute, Cedars-Sinai Medical Center, Los Angeles, CA 90048 USA; 3 Wellcome Trust Sanger Institute, Wellcome Genome Campus, Hinxton, CB10 1SA UK; 4 European Bioinformatics Institute, Wellcome Genome Campus, Hinxton, CB10 1SD UK; 5 Department of Biochemistry, MU Metabolomics Center, University of Missouri Bond Life Sciences Center, Columbia, MO 65201 USA; 6 Mutant Mouse Resource and Research Center, University of Missouri, 4011 Discovery Drive, Columbia, MO 65201 USA

**Keywords:** Genetics, Gut microbiota, Colorectal cancer, Metabolomics, Apc, Min

## Abstract

**Background:**

Colorectal cancer (CRC) is a multifactorial disease resulting from both genetic predisposition and environmental factors including the gut microbiota (GM), but deciphering the influence of genetic variants, environmental variables, and interactions with the GM is exceedingly difficult. We previously observed significant differences in intestinal adenoma multiplicity between C57BL/6 J-*Apc*^*Min*^ (B6-*Min*/J) from The Jackson Laboratory (JAX), and original founder strain C57BL/6JD-*Apc*^*Min*^ (B6-*Min/*D) from the University of Wisconsin.

**Methods:**

To resolve genetic and environmental interactions and determine their contributions we utilized two genetically inbred, independently isolated *Apc*^*Min*^ mouse colonies that have been separated for over 20 generations. Whole genome sequencing was used to identify genetic variants unique to the two substrains. To determine the influence of genetic variants and the impact of differences in the GM on phenotypic variability, we used complex microbiota targeted rederivation to generate two *Apc* mutant mouse colonies harboring complex GMs from two different sources (GMJAX originally from JAX or GMHSD originally from Envigo), creating four *Apc*^*Min*^ groups. Untargeted metabolomics were used to characterize shifts in the fecal metabolite profile based on genetic variation and differences in the GM.

**Results:**

WGS revealed several thousand high quality variants unique to the two substrains. No homozygous variants were present in coding regions, with the vast majority of variants residing in noncoding regions. Host genetic divergence between *Min*/J and *Min*/D and the complex GM additively determined differential adenoma susceptibility. Untargeted metabolomics revealed that both genetic lineage and the GM collectively determined the fecal metabolite profile, and that each differentially regulates bile acid (BA) metabolism. Metabolomics pathway analysis facilitated identification of a functionally relevant private noncoding variant associated with the bile acid transporter *Fatty acid binding protein 6* (*Fabp6*). Expression studies demonstrated differential expression of *Fabp6* between *Min*/J and *Min*/D, and the variant correlates with adenoma multiplicity in backcrossed mice.

**Conclusions:**

We found that both genetic variation and differences in microbiota influences the quantitiative adenoma phenotype in *Apc*^Min^ mice. These findings demonstrate how the use of metabolomics datasets can aid as a functional genomic tool, and furthermore illustrate the power of a multi-omics approach to dissect complex disease susceptibility of noncoding variants.

## Background

Colorectal cancer (CRC) is a complex disease trait resulting from a variety of factors including genetic predisposition, diet, age, inflammation, and lifestyle [1–3]. Malignant disease is preceded by the initiation of adenomas in the epithelial lining of the intestinal mucosa, and often persist up to 10 years before acquiring malignant transformations, making the adenoma a critical target for early intervention [4]. Recently, CRC has been associated with perturbations in the gut microbiota (GM) through postulated mechanisms including modulation of inflammation, genotoxin production, and metabolic homeostasis [5–8], but it is often unclear whether these shifts in bacterial composition directly impact disease risk, or merely result from physiological changes associated with disease. Initiation and progression of adenomas is likely determined by a combination of genetic factors and changes in microbial populations that mutually impact relevant pathways [9]. However, the ability to successfully integrate these complex factors and to dissect the independent and additive effects of each remains elusive in human populations.

The intestinal environment is collectively comprised of dynamic interactions between diet, modified host compounds, and microbial metabolites [10]. As such, changes in host functional genomic output via germline or acquired mutations, or shifts in the functional GM, may substantially influence the metabolite profile. Using metabolomics provides an avenue to interrogate the metabolic output of complex biological systems in a non-targeted discovery-based approach [11]. In controlled experiments, metabolites represent a highly sensitive means of detecting functional changes associated with genomic variation, differences in complex microbial communities, and even more importantly the combination of these factors in the context of complex disease traits. Several studies have demonstrated the utility of characterizing metabolite profiles in colorectal cancer, identifying microbial metabolites including short-chain fatty acids (SCFAs) such as butyrate that can influence gene expression, cell proliferation, and ultimately adenoma formation [12]. Furthermore, altered levels of microbial-influenced metabolites including bile acids (BA) and hydrogen sulfide (H_2_S) are associated with both inflammatory bowel disease and CRC through the production of genotoxic reactive oxygen species [8, 13–15]. As an approach, non-targeted metabolomics data correlate to 16S rRNA microbiome composition more strongly than targeted metabolomics, and have identified novel metabolites in CRC patients [16].

Due to the challenges of controlling environmental conditions and performing longitudinal monitoring of disease progression from pre-disease stages in human populations, adequate models need to be refined to study early initiating events. The *Apc*^*Min*^ (Min) mouse model of CRC, which harbors an autosomal dominant mutation in the *Apc* tumor suppressor gene causing the development of intestinal adenomas, provides an extensively studied platform to interrogate genomic and GM contributions to disease initiation in a quantitative manner [17]. Investigators using this model have observed complex genetic modification of the adenoma phenotype from multiple modifier genes, including modifiers between mouse strains and newly arising variants within the C57BL/6 J strain [18–20]. It is now clear that in addition to both known and unknown genetic factors, the GM can also impact adenoma initiation and progression, as germ-free Min mice develop significantly lower adenoma burdens than their colonized counterparts [21]. Still, it is unclear how functional genomic changes and distinct GM communities independently and additively influence adenoma initiation in the context of the complex specific-pathogen-free GM. Thus, the Min mouse provides a platform to dissect genomic and microbial contribution to phenotypic variability, and draw further inferences about variable disease susceptibility across human populations.

A small sampling of the tumor count data reported in the Min mouse shows a wide range of small intestinal tumor counts among control animals. Throughout the course of over two decades of use of the C57BL/6 J-*Apc*^*+/Min*^ mouse, reported adenoma counts across different colonies have varied substantially (Table 1). In some cases, these disparities were attributed to undetermined differences between institutions. It is well-established that mice originating from different mouse producers and institutions have highly distinct GMs [22]. Furthermore, strict genetic control of mouse models is essential to maintaining a consistent phenotype. Though producers take precautions to prevent genetic drift in inbred colonies, mutations in genetic modifiers of the Min phenotype may be selected for rapidly within a colony, and thus account for differences in tumor number across different colonies. In this study, we leveraged the observed phenotypic variability between two inbred Min colonies from a common lineage that have been separated in excess of 20 generations, to interrogate whether disparity in tumor numbers between C57BL/6 inbred colonies occurs due to differences in the GM or host genetic differences associated with colony divergence. We transferred embryos from mice from a low-tumor multiplicity colony (C57BL/6 J-*Apc*^*Min*^/J abbrv. *Min*/J) and a high-tumor multiplicity colony (C57BL/6JMlcr-*Apc*^*Min*^/Mlcr abbrv. *Min*/D) into surrogate dams harboring distinct complex GMs, resulting in two genetically independent lines of mice each harboring two distinct complex GMs. We describe independent and additive influences of host genetics and the GM on adenoma initiation through the use of 16S rRNA microbial profiling, host whole-genome sequencing (WGS), and finally non-targeted metabolomics. This approach allows for the relatively non-invasive identification of altered biologically relevant pathways and mechanistic associations with CRC initiation through integration and refinement of large data sets.

**Table 1:** Summary of small intestinal (SI) adenoma number variability between C57BL6/J-*Apc*^*Min*^ colonies

Tumor Count (SI)	Reference
22	MacGregor DJ et al. International Journal of Oncology. 2000.
34	Zell JA et al. International Journal of Cancer. 2007.
41	Chiu CH et al. Cancer Research. 1997.
71	Niho N et al. Cancer Science. 2003.
102	Ahn B and Ohshima H. Cancer Research. 2001.
108	Paulsen JE et al. Carcinogenesis. 1997.
128	Kwong et al. Genetics. 2007.

## Methods

### Animal use and ethics statement

**Fig. 1:**
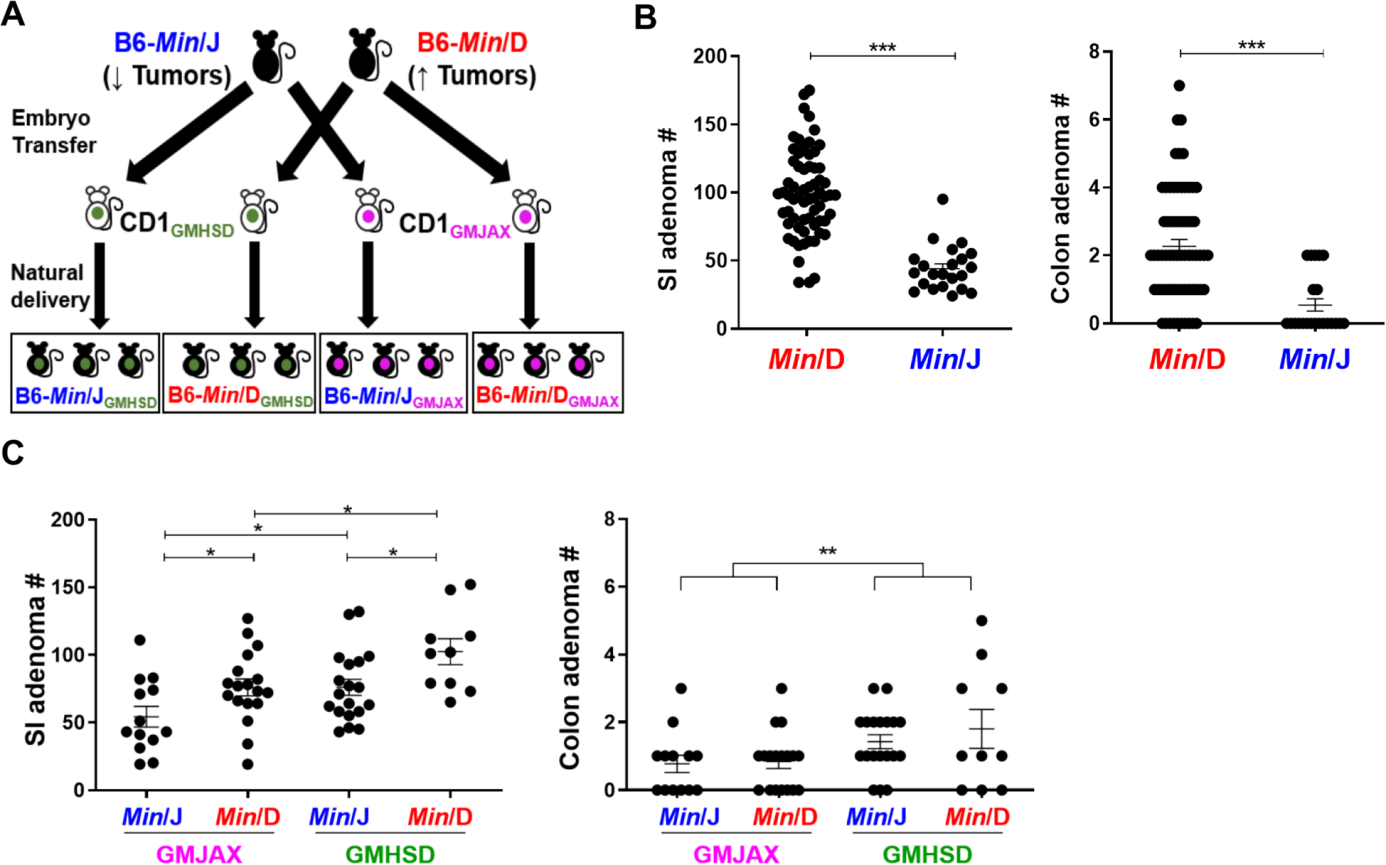
Genetic lineage and GM colonization additively determine adenoma numbers in ApcMin mice. **a** Embryos from the *Min*/J and *Min*/D genetic lineages were transplanted into surrogate dams harboring two distinct complex GM profiles; GMJAX and GMHSD. Offspring represent the two genetic lineages which have inherited a GM from their respective surrogate dams (*Min*/J_GMJAX,_*n* = 13; *Min*/D_GMJAX,_*n* = 18; *Min*/J_GMHSD,_*n* = 19; *Min*/D_GMHSD_, *n* = 10). **b** Scatter plots comparing mean (± SEM) small intestinal (SI) and colon adenoma counts of the original *B6-Apc*^*Min*^ colony generated at UW McArdle Laboratory (Min/D) to *B6-Apc*^*Min*^ mice acquired from The Jackson Laboratory and maintained at University of Missouri (*Min*/J) (*Min*/D, *n* = 65; *Min*/J, *n* = 22). **c** Scatter plots comparing mean (± SEM) SI and colon adenoma counts of the four rederived groups, including each genetic lineage (*Min*/J and *Min*/D) rederived with two complex GMs. *p < 0.05, **p < 0.01, ***P < 0.001; Student’s t-test (**a**) and Two-way ANOVA with the Student Newman-Keuls method (**c**)

Animal studies were conducted in an Association for Assessment and Accreditation of Laboratory Animal Care International (AAALAC) accredited facility according to the guidelines provided by the Guide for the Care and Use of Laboratory Animals, and were approved by the University of Missouri Institutional Animal Care and Use Committee. For Complex Microbiota Targeted Rederivation (CMTR) C57BL/6JMlcr*-Apc*^*Min*^/Mmmh (*Min*/D) and C57BL/6 J-*Apc*^*Min*^/J (*Min*/J) embryos were transferred into separate Crl:CD1 surrogate dams with distinct complex GM populations (GMJAX and GMHSD) to naturally deliver offspring representing four experimental groups; *Min*/J_GMJAX_, *Min*/D_GMJAX_, *Min*/J_GMHSD_, and *Min*/D_GMHSD_ (Fig. 1a).

Male and female CMTR offspring were group-housed by sex, genetic origin of the embryo donor (*Min*/D or *Min*/J), and GM of the surrogate dam (GMJAX or GMHSD). All mice, including embryo donors, ET recipients, and rederived offspring were group-housed in microisolator cages on ventilated racks (Thoren, Hazelton, PA) on a 14:10 light:dark cycle on paper chip bedding (Shepherd Specialty Papers, Watertown, TN), with ad libitum access to 5058 irradiated breeder chow (LabDiet, St. Louis, MO) and acidified autoclaved water. All pups were ear-punched at weaning (21 days of age) using sterile technique. DNA was extracted using the “HotSHOT” genomic DNA preparation method as described [23]. To generate N2 backcross animals *Min*/D males and WT females from the *Min*/J colony were first crossed to create F1 hybrids of the two genetic lineages. F1 hybrids were then backcrossed to both the *Min*/D and *Min*/J parental lines to create N2 mice. At 3 months of age, all mice were euthanized via CO_2_ asphyxiation and the abdominal cavity was opened. Whole small and large intestines were incised longitudinally, flushed with saline and placed on bibulous paper with the luminal side facing up for formalin fixation. Grossly visible adenomas were counted manually using a Leica M165FC microscope at 1.25x magnification. Fecal samples were collected from all rederived mice at 1 month, while fecal samples, cecal material, and ileal scrapes were collected after sacrifice at 3 months of age.

### Embryo collection and transfer

Embryos for transfer were collected from donors from two separate colonies (ET donors). Half of the embryos were obtained from frozen stocks that were generated through breeding of sexually mature C57BL/6JD-*Apc*^+/*Min*^ (*Min*/D) males with 5–8 week-old C57BL/6JD*-Apc*^+/+^ females, maintained as a closed-colony at the McArdle Laboratory, University of Wisconsin (Madison, WI). A second cohort of embryos for ET was obtained on-site (University of Missouri, Columbia, MO) using C57BL/6 J-*Apc*^+/*Min*^ (*Min*/J) males and 5 to 8 week-old C57BL/6 J- *Apc*^+/+^ females, purchased from The Jackson Laboratory (Bar Harbor, ME). To generate *Min*/J embryos, in vitro fertilization was performed as described [24]. Presumptive zygotes were then moved to a KSOM dish and incubated for 24 h to allow progression to the two-cell stage [25]. For ET recipients, 8 week old CD1 females harboring a GM (Hsd:CD1_GMHSD_) from Envigo (Envigo, Indianapolis, IN) were purchased and allowed to acclimate for 1 week prior to use. Eight week old CD1 females harboring a GM representing The Jackson Laboratory (Crl:CD1_GMJAX_) were previously generated in our laboratory [26]. CD1_GMHSD_ and CD1_GMJAX_ surrogate female embryo recipients were mated with sterile, vasectomized Hsd:CD1 or Crl:CD1 males, respectively. All surrogate females were inspected for copulatory plugs and plug-positive mice were used for embryo transfer. Surrogate females were anesthetized via IM injection of ketamine/xylazine cocktail at 5.5 mg and 1 mg per 100 g body weight respectively, and placed in sternal recumbency. A dorsal midline incision was made and the uterine oviducts located by dissecting through the retroperitoneal muscle. Embryos in 3 to 5 μl of media were injected into the oviducts using a glass hand-pipette. Skin incisions were closed with sterile surgical staples and mice received a subcutaneous injection of 2.5 mg/kg of body weight flunixin meglumine (Banamine®) prior to recovery on a warming pad.

### Tissue collection and processing

All mice were humanely euthanized with CO_2_ asphyxiation and necropsied, and small intestines were processed as described above. A sterile scalpel blade was used to gently scrape normal ileal epithelium. After the body cavity was opened, whole spleens and liver were also collected. All collected tissue was flash-frozen in liquid nitrogen followed by storage at − 80 °C.

### Sample collection and DNA extraction for 16S rRNA sequencing

Two fecal pellets per mouse were collected aseptically and placed in a 2 mL round-bottom tube containing 800 μl of lysis buffer [22] and a 0.5 cm diameter stainless steel bead (Grainger, Lake Forest, Il). All samples were mechanically disrupted using a TissueLyser II (Qiagen, Venlo, Netherlands) for 2 min at 50 Hz, followed by incubation at 70 °C for 20 min with periodic vortexing. DNA extraction from fecal pellets, cecal contents, and ileal epithelium for 16S rRNA sequencing was performed using a DNeasy Blood & Tissue Kit® (Qiagen) as previously described [22].

### 16S library preparation and sequencing

DNA extraction from fecal pellets, cecal contents, and ileal epithelium for 16S rRNA sequencing was performed using a DNeasy Blood & Tissue Kit® (Qiagen) as previously described (See Supplemental Methods) [22]. Bacterial 16S rRNA amplicons were generated by amplification of the V4 hypervariable region of the 16S rRNA gene using universal primers (U515F/806R) [27], then sequenced on the Illumina MiSeq platform as described previously [22]. Assembly, binning, and annotation of DNA sequences was performed using Qiime v1.9 [28] at the University of Missouri Informatics Research Core Facility (Columbia, MO) as described [22]. Contiguous sequences were assigned to operational taxonomic units (OTUs) using a criterion of 97% nucleotide identity by de novo clustering. Taxonomy was assigned to selected OTUs using BLAST [29] against the SILVA database [30] of 16 s rRNA sequences and taxonomy.

### Whole-genome sequencing

Genomic DNA for whole-genome sequencing (WGS) was extracted from splenic tissue using the DNeasy Blood & Tissue Kit®, as described by the manufacturers (Qiagen). Paired-end (151 base pair) sequence reads generated for each sample were aligned to the GRCm38 (mm10) mouse reference genome using BWA-MEM (v0.7.5) [http://arxiv.org/abs/1303.3997] followed by a local realignment around indels using the GATKv3.0 ‘IndelRealigner Tool’ [20644199]. Possible PCR and optical duplicates were filtered using Picard tools (v1.64) (http://broadinstitute.github.org/picard). SNP and short indel calls were generated using the Mouse Genomes Project variation catalog v5 parameters (described in detail [27480531]). In brief, samtools mpileup v1.3 [19505943] and bcftools call v1.3 [21653522] were used to identify SNPs and indels in each of the samples. Indels were left-aligned using the bcftools norm function. Filters were then applied to removed variants of low depth (< 10 reads), low genotype quality (q < 20), poor mapping quality (q < 20) and proximity to an indel (SNPs within 2 bp of an indel). Additionally, only heterozygous SNPs with > 5 support reads for each allele were retained. Functional consequences based on mouse Ensembl gene models (v88) were annotated using the Variant Effect Predictor [20562413]. The VEP tool facilitates the identification of synonymous and deleterious mutations such as stop changes and potentially damaging missense variants. Variants private to each sample were identified by removing SNPs and indels common to any of the 36 strains present in the MGPv5 catalog [27480531].

### TA cloning and sanger sequencing for variant validation

As described previously in *Genotyping*, ear punches were used to collect DNA for variant validation. To validate the observed variant in the upstream region of *Fabp6* detected by WGS, this region was PCR amplified using the primers FWD 5′-ACCACTTCCTCCCTCAGGAT-3′, REV 5′-TTCTCCCAATGCCCATCCAG-3′. The TOPO TA Cloning® Kit (Invitrogen™) was used to insert the region of interest into the pCR™ 4-TOPO® vector, and TOP10 competent *E. coli* cells were used for vector transformation according to the manufacturer’s instructions. Transformed cells were spread onto Lysogeny Broth (LB) plates with 50 μg/mL kanamycin for resistance selection, then grown overnight at 37 °C in a shaking incubator. The PureYield™ Plasmid Miniprep System (Promega, Madison, WI) was used to extract DNA from each culture according to the manufacturer’s instructions. Sequencing reactions were prepared using the extracted DNA and the T7 sequencing primer (5′-TAATACGACTCACTATAGGG-3′. Sanger sequencing was performed at the MU DNA Core using a 3730xl 96-capillary DNA analyzer (ThermoFisher Scientific, Waltham, MA) with the Applied Biosystems Big Dye Terminator cycle sequencing chemistry.

### Genotyping

Genotyping for the Min allele by PCR was performed in a reaction volume of 10 uL containing 0.2 uM of each primer (5′-ATTGCCCAGCTCTTCTTCCT-3′ and 5′-CGTCCTGGGAGGTATGAATG-3′), 1 x HRM Supermix (BioRad, Hercules, CA), and genomic DNA. Genotyping for the *Fabp6* upstream insertion was similarly performed using ear-punches as described. The 10 uL HRM reaction contained 0.2 uM of each primer (5′-ACCACTTCCTCCCTCAGGAT-3′ and 5′-TTCTCCCAATGCCCATCCAG-3′), 1 x HRM Supermix, and genomic DNA. Genotyping reactions and analyses were carried out using a BioRad CFX384 Real-Time PCR Detection system. For Min genotyping, cycling conditions were as follows: 95 °C, 2 min; 40 cycles of 95 °C, 10 s; 60 °C, 30 s, 72 °C, 30 s, 95 °C, 30 s; 60 °C, 1 min, followed by melt curve analysis from 73 °C to 85 °C in increments of 0.1 °C for 10 s. PCR cycling conditions for *Fabp6* analysis were the same as those mentioned above, followed by a melt curve analysis from 65 °C to 95 °C in increments of 0.2 °C. All melt curve results were analyzed using BioRad Precision Melt Software v1.2 to detect the Min allele or the *Fabp6* insertion.

### Tissue processing and reverse transcriptase-quantitative PCR (RT-qPCR)

Ileal scrapes collected at 3 months of age were used to quantitate expression of *Fabp6*, and liver used to quantitate expression of *Cyp39a1*. All collected tissue was flash-frozen in liquid nitrogen followed by storage at − 80 °C. Frozen tissues were mechanically disrupted using a TissueLyser II (Qiagen) for 4 min at 50 Hz. Total RNA was then extracted using the AllPrep® DNA/RNA Mini Kit (Qiagen), and cDNA was synthesized using the iScript™ cDNA Synthesis Kit (Bio-Rad, Hercules, CA) according to the respective manufacturer’s instructions. Samples were analyzed in quadruplicate and all evaluated gene expression levels were normalized to *Hprt* expression using a PrimeTime® qPCR assay (IDT®). For qPCR, each 10 uL reaction contained 1 x Primer/Probe mixes (Table S9), 1 x iTaq™ Universal Probe Supermix, and 100 ng cDNA template. PCR parameters were: denaturation at 95 °C for 5 s, and annealing and elongation at 60 °C for 30 s for a total of 54 cycles.

### Ultra-high performance liquid chromatography-tandem mass spectrometry (UHPLC-MS/MS)

Fecal samples weighing 25 mg were treated with 1.0 mL 80% MeOH with 18 μg/mL umbelliferone, sonicated for 5 min and centrifuged for 40 min at 3000 g at 10 °C. 0.5 mL supernatant was used for UHPLC-MS analysis after centrifugation at 5000 g at 10 °C for 20 min and transfer of 250 μL of extract into glass vials with inserts. A Bruker maXis impact quadrupole-time-of-flight mass spectrometer coupled to a Waters ACQUITY UPLC system was used to perform UHPLC-MS analysis. Compound separation was achieved on a Waters C18 column (2.1 × 100 mm, BEH C18 column with 1.7-um particles) using a linear gradient and mobile phase A (0.1% formic acid) and B (acetonitrile). Phase B increased from 5 to 70% over 30 min, then to 95% over 3 min, held at 95% for 3 min, then returned to 5% for equilibrium. Flow rate was 0.56 mL/min and the column temperature was 60 °C. Mass spectrometry was performed in the negative electrospray ionization mode with the nebulization gas pressure at 43.5 psi, dry gas of 12 l/min, dry temperature of 250 C and a capillary voltage of 4000 V. Mass spectral data were collected from 100 and 1500 m/z and were auto-calibrated using sodium formate after data acquisition. Instrument performance was monitored by the internal standard umbelliferone and peak areas of metabolites were normalized to the internal standard. One sample from each of the four experimental groups was analyzed with automated MS/MS. Fragmentation data was compared to archived PUBCHEM and KEGG fragment databases via the MetFrag web tool (https://msbi.ipb-halle.de/MetFragBeta/).

### Metabolomics data analysis

Chromatographic data was aligned using mass and retention time with XCMS software (http://xcmsonline.scripps.edu/). Following alignment, XCMS was used to generate a relative intensity table with individual features labeled by retention time and mass for analysis in the Metaboanalyst v3.0 web program [31]. In Metaboanalyst, the interquartile range method was used to filter data. Data was normalized based on sample sums of features’ relative intensity, then log transformed prior to multivariate analysis. Principle Component Analysis (PCA), putative metabolite identification, and pathway overrepresentation cloud plots were generated with XCMS, where dysregulated pathways were determined using the mummichog algorithm [32]. Metaboanalyst was used to perform hierarchical clustering using the Euclidean distance measure and Ward clustering algorithm with significantly modulated (based on ANOVA) metabolites according to experimental group, and displayed as a heat-map and dendogram. Metabolite and tumor correlation analysis was performed using small intestinal tumor counts and individual feature relative intensities across all four experimental groups, and regression graphs were generated using GraphPad Prism 8. Individually significant features were determined separately in terms of GM (compared *Min*/D_GMJAX_ and *Min*/D_GMHSD_) and genetic lineage (compared of *Min*/J_GMJAX_ and *Min*/D_GMJAX_) by t-test in XCMS. To determine the metabolites contributing to the separation and rooting of the hierarchical clusters illustrated by the dendogram, the samples were classified into those with ‘high’ or ‘low’ colonic adenoma numbers independent of genetic lineage or GM, and a linear discriminant analysis (LDA) was performed using the LEfSe (Linear discriminant analysis Effect Size) tool on a high-computing Linux platform [33]. An LDA score of log_10_2 or greater for any given metabolite was considered significantly differential between the high and low adenoma groups.

### Statistical analysis

Statistical analyses were performed using Sigma Plot 14.0 (Systat Software Inc., Carlsbad CA). Differences in OTU relative abundance between GMJAX and GMHSD were determined using Student’s t-test. To account for multiple testing, OTUs with a *p* value < 0.001 were considered statistically significant. Two-way ANOVA with the Student Newman-Keuls post-hoc method was used to assess differences in adenoma number between rederived groups, where *p* < 0.05 was considered statistically significant. For GM analysis, GraphPad Prism 8 was used to generate bar graphs and Tukey’s box plots displaying phylum relative abundances, richness (OTU counts), and α-diversity (Shannon Index). Principal Coordinate Analyses incorporating the Bray-Curtis similarity index used for visualizing β-diversity were generated with the Paleontological Statistics software package (PAST) 3.12 [34]. Two-way ANOVA/Student Newman-Keuls post-hoc method was used to assess differences in richness and α-diversity and phylum abundance differences between rederived mice. To better account for quantitative and qualitative community differences between GMJAX and GMHSD, statistical testing for β-diversity was performed via a two-way PERMANOVA analysis of both Bray-Curtis and Jaccard dissimilarities for bacterial OTU community structure using PAST 3.12. For RT-qPCR assays, expression analysis was performed using the 2^-ΔΔCt^ method of relative expression [35], and statistical differences were assessed using the Student’s t-test.

## Results

### Genetic lineage and GM colonization additively determine adenoma susceptibility in distinct C57BL/6-*Apc*^*Min*^ colonies

Historically, tumor multiplicities in C57BL/6-*Apc*^*Min*^ mice vary widely in reported studies despite having the same inbred genetic background (Table 1). Notably, these colonies were housed in different institutions for unknown numbers of generations prior to reporting tumor numbers, highlighting the difficulty in separating the impact of genetic divergence from environmental variables. We compared intestinal adenoma number in our institution between two C57BL/6-*Apc*^*Min*^ lines arising from a common colony. The original B6-*Apc*^*Min*^ colony was developed in the McArdle Laboratory at the University of Wisconsin (*Min*/D). A subset of *Min*/D mice were sent to the Jackson Laboratory (JAX) and underwent rederivation for colony development and distribution (*Min*/J), and thus harbor a GM representing JAX. The *original Min*/D colony was maintained as a closed colony through sibling mating and harbored a GM from Harlan/Sprague Dawley (now Envigo) that was acquired through pup fostering to ICR (Hsd:ICR (CD-1)) foster mice to rid the colony of *Helicobacter* spp. Mice from the *Min*/D colony had an average of 99.2 small intestinal (SI) and 2.26 colonic adenomas [36], and breeder males were consistently progeny-tested to maintain tumor multiplicities in the offspring within one standard deviation from the average. The *Min*/J colony acquired from the Jackson Laboratory and maintained at the University of Missouri had significantly fewer SI and colonic adenomas, with 44.2 and 0.55, respectively (SI and colon *p* < 0.001) (Fig. 1a).

To interrogate how GM and host genetic lineage independently and additively contribute to variable adenoma susceptibility in *Apc*^*Min*^ mice, we used Complex Microbiota Targeted Rederivation (CMTR) to establish mice from the *Min*/J genetic lineage and the *Min*/D genetic lineage with two different complex GMs; a low-richness GM originally acquired from B6 mice from the Jackson Laboratory (GMJAX) and high-richness GM originally acquired from CD-1 mice from Envigo (GMHSD). These GM profiles were chosen because they most closely represent the original GMs of the *Min*/J and *Min*/D colonies, respectively. *Min*/J and *Min*/D embryos were separately implanted into surrogate dams harboring the desired GM, such that they would maintain their original genetic lineage while acquiring the desired maternal GM through natural birth. Thus, we generated four experimental groups representing each combination of genetic lineage and GM (Fig. 1b). All *Apc*^*Min*^ offspring were sacrificed at 3 months of age, and SI and colonic adenomas were counted to determine the effects of genetic lineage and GM colonization on adenoma susceptibility. We found that independent of genetic lineage, mice colonized with GMHSD developed more SI adenomas than their GMJAX counterparts. Furthermore, when comparing adenoma susceptibility between the genetic lineages within each GM, mice of the *Min*/D lineage developed more adenomas than *Min*/J mice independent of GM (Fig. 1c). Thus, colonization of *Min*/J embryos with GMHSD partially restored the original *Min*/D phenotype, but did not account entirely for the phenotypic differences between the original *Min*/D and *Min*/J colonies. Colonization of *Min*/D embryos with GMJAX suppressed the original *Min*/D phenotype, while colonization of *Min*/D with GMHSD completely restored the original McArdle phenotype. Combining the effects of genetic lineage and GM, *Min*/D_GMHSD_ mice develop substantially more adenomas than *Min*/J_GMJAX_ (*p* < 0.001). In the colon, we observed increased adenomas in GMHSD-colonized mice compared to GMJAX, while genetic lineage had no apparent effect (Fig. 1c). These trends were similarly observed when males and females were assessed separately (Fig. S1). To summarize, both genetic lineage and GM colonization independently modulated adenoma susceptibility, and collectively had either additive protective or deleterious phenotypic effects.

### Distinct GM communities influence adenoma susceptibility

**Fig. 2:**
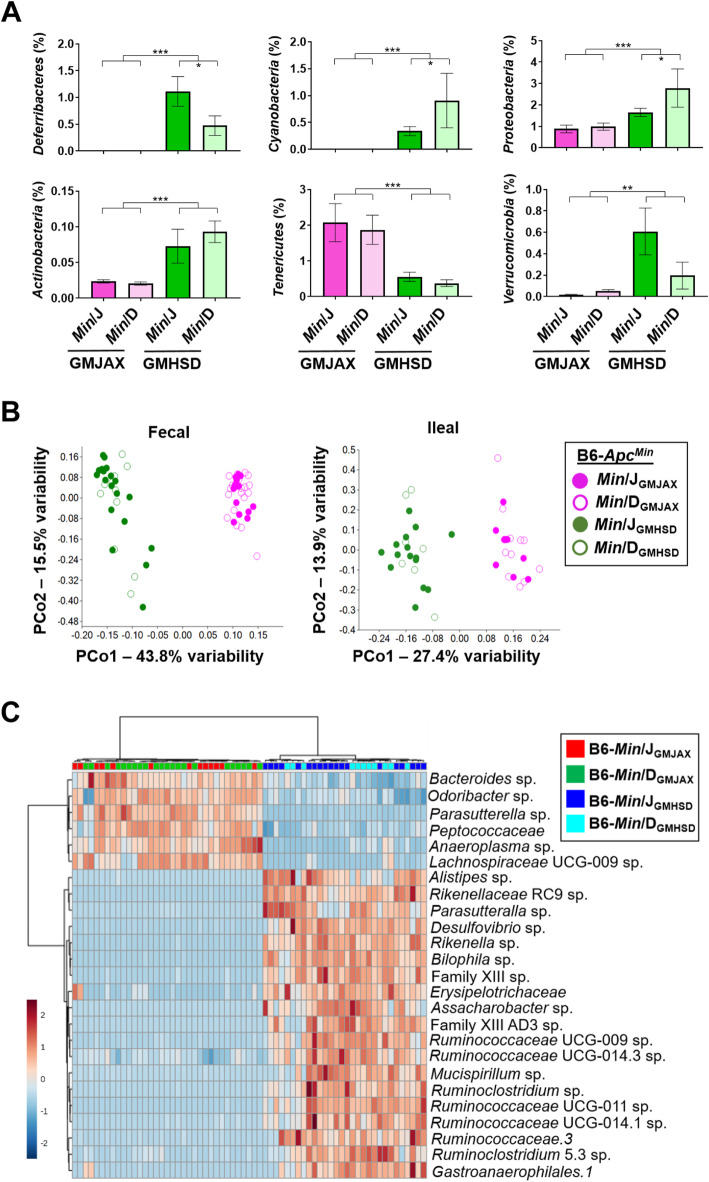
Distinct GM communities influence adenoma susceptibility. **a** Bar charts representing relative abundances (mean ± SEM) of Phyla with detected significant differences between fecal samples GMJAX and GMHSD groups (*Min*/J_GMJAX,_*n* = 13; *Min*/D_GMJAX,_*n* = 18; *Min*/J_GMHSD,_*n* = 19; *Min*/D_GMHSD_, *n* = 10). **b** Unweighted Principal Coordinate Analysis (PCoA) representing differences in β-diversity at the Operational Taxanomic Unit (OTU) level between complex GM profiles of CMTR offspring in feces at 1 month, and ileal scrapes at 3 months of age. **c** Heatmap showing 25 taxa with significantly different (*p* < 0.001) fecal relative abundances between GMJAX and GMHSD at 1 month, where color intensity represents fold-change of each OTU. Hierarchical clustering based on Euclidean distances (top) demonstrates clustering of samples based on GM. All statistically significant OTUs and associated log-fold changes are represented in supplementary Tables 3A (fecal) and 3B (ileal).*p < 0.05, **p < 0.01, ***p < 0.001; Two-way ANOVA with the Student Newman-Keuls method for Multiple Comparisons

To characterize the GMJAX and GMHSD microbial communities, feces were collected at 1 month, and fecal and ileal epithelial scrapes at 3 months of age, from rederived *Apc*^*Min*^ mice. 16S rRNA sequencing was then used to determine relative abundance of all detected microbial taxa. At 1 month, phyla *Proteobacteria, Actinobacteria*, *Deferribacteres,* and *Cyanobacteria* were enriched in GMHSD-colonized mice, while *Tenericutes* were enriched in GMJAX-colonized mice (Fig. 2a). These changes were observed regardless of genetic lineage, indicating that phylum make-up was determined by the surrogate dam rather than genetic lineage of the embryo. At the operational taxonomic unit (OTU) level, GMJAX and GMHSD had distinct post-weaning microbial profiles in fecal samples (Fig. 2b) which remained disparate until sacrifice at 3 months in both feces and ileal scrapes (Fig. S2A). Community analyses of fecal and ileal β-diversity by two-way PERMANOVA corroborated the discrete nature of these communities (Table S1 and S2). Sex did not appear to play a significant role in GM make-up, and as anticipated based on previous characterization of these GMs [22], GMHSD mice had increased microbial richness (Chao1 index) and α-diversity (Shannon Index) compared to GMJAX mice (Figs. S2B and S2C). Using a *p*-value of 0.001 as a threshold, we found 58 and 34 significantly modulated OTUs in feces and ileal scrapes, respectively, between GMJAX and GMHSD (Tables S3 and S4). GMHSD mice harbored enriched abundances of sulfidogenic *Desulfovibrio* and *Bilophila* sp., as well as sulfatase-secreting bacteria (SSB) *Rikenella*, while GMJAX had enriched levels of *Bacteroides* sp. and family *Peptococcaceae*. A heat map illustrating fold difference in the relative abundance of the 25 most significantly different OTUs was used for a hierarchical clustering analysis, and shows that samples clustered based on GM profile, regardless of genetic lineage (Fig. 2c). Thus, GMJAX and GMHSD represent highly distinct complex microbial communities with a number of different taxa potentially contributing to differential adenoma susceptibility.

### GM and host genetic lineage shape the metabolome in *Apc*^*Min*^ mice

**Fig. 3:**
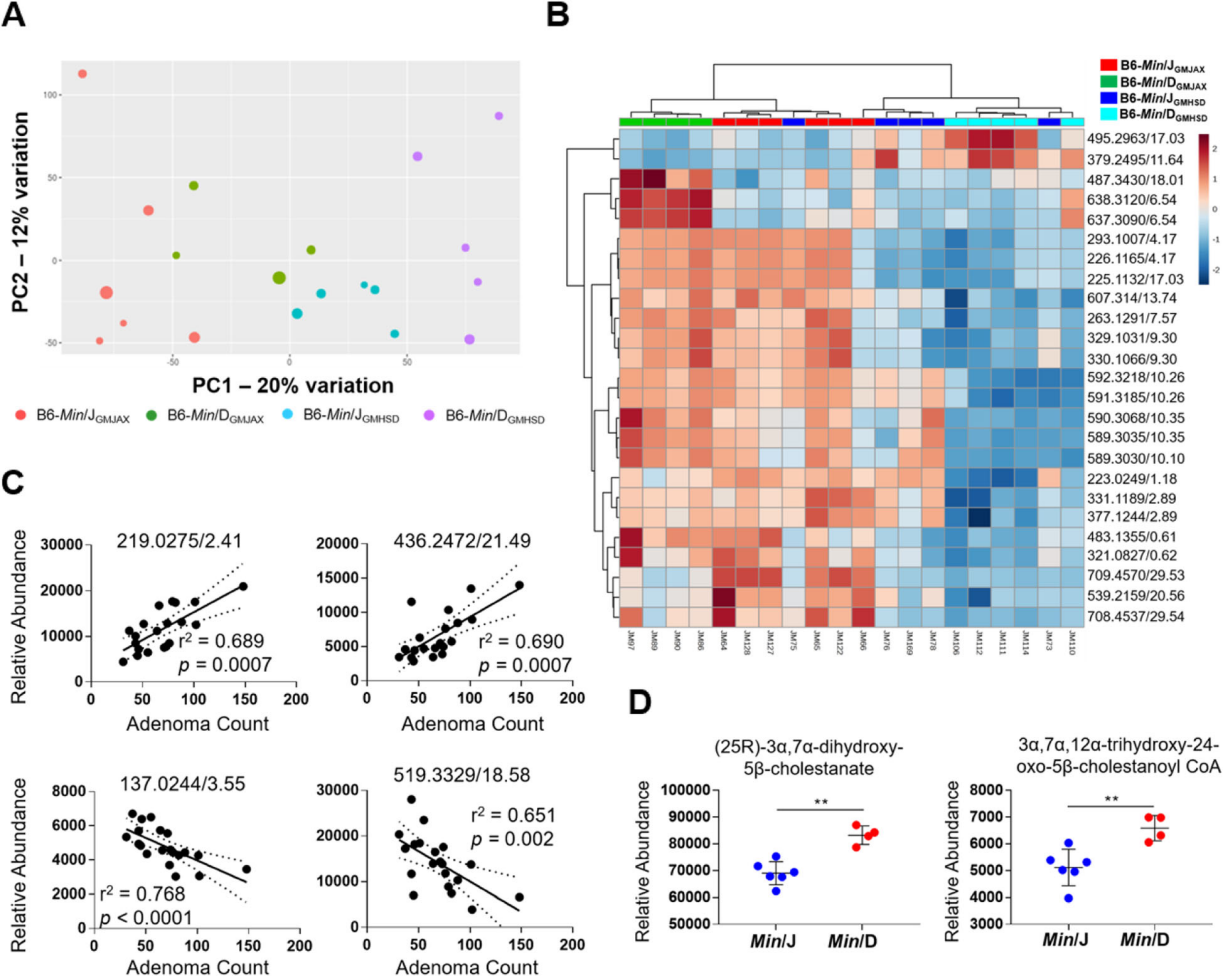
Untargeted analysis of GM and host genetic lineage effects on the fecal metabolome. **a** PCA illustrating unsupervised clustering of fecal metabolites at 3 months of age (*Min*/J_GMJAX,_*n* = 6; *Min*/D_GMJAX,_*n* = 4; *Min*/J_GMHSD,_*n* = 5; *Min*/D_GMHSD_, *n* = 5). **b** Heatmap showing 25 detected fecal metabolites with most significantly different relative abundances across all rederived groups, where color intensity represents log-fold-change of each metabolite. Hierarchical clustering based on Euclidean distances (top) illustrates primary clustering of samples based on GM, and secondary clustering based on genetic lineage. All metabolites shown on heat map have significantly different mean abundances (*p* < 0.001) based on ANOVA. **c** Spearman’s rank correlation was used to show metabolites with significant positive or negative correlations to SI tumor number across all rederived *Apc*^*Min*^ groups (*n* = 20). **d** Scatter plots of mean ± SEM relative abundances of putative metabolites contributing to modulation of bile acid metabolism (*Min*/J, *n* = 6; *Min*/D, *n* = 4). Metabolites are denoted by mass:charge ratio and retention time (m/z_t_R_). *p < 0.05, **p < 0.01, ***p < 0.001; Student’s t-test

Based on the results of our rederivation experiment, we aimed to determine functional differences between each genetic lineage and GM community that could contribute to differential disease susceptibility using a metabolomics approach. Feces contains not only microbial metabolites, but also mammalian host metabolites that may undergo microbial biotransformation [10]. In an untargeted analysis of fecal metabolites at 3 months of age detected by liquid chromatography coupled mass spectrometry (LC-MS), we observed distinct metabolite profiles based on both genetic lineage and GM colonization (Fig. 3a). Using a False Discovery Rate (q-value) of 0.1 as a threshold, we found that 1009 features were significantly modulated between the four rederived *Apc*^*Min*^ groups. Of these features, 172 were specifically modulated by the GM and 7 by genetic lineage (Supplementary datasets 1-3; Figs. S3A and B), while the remainder appear to be modulated by a combination of the two factors. A heat map illustrating fold-change of the most substantially modulated metabolites (based on ANOVA) was used for a hierarchical clustering analysis. This analysis demonstrated that samples primarily clustered based on GM, with a secondary clustering pattern based on genetic lineage (Fig. 3b). Notably, we found that certain metabolites had significant positive and negative correlations with adenoma number across all four rederived groups (Fig. 3c). A pathway analysis using putative compounds was performed to determine metabolic pathways modulated based on genetic lineage and GM colonization. Differential bile acid metabolism was observed when comparing *Min*/J and *Min*/D genetics, as defined by enrichment of putative bile acid intermediates (25R)-3α,7α-dihydroxy-5β-cholestanate and 3α,7α,12α-trihydroxy-24-oxo-5β-cholestanoyl CoA in *Min*/D mice compared to *Min*/J (Table S5, Fig. 3d). Meanwhile, differential sphingosine lipid metabolism was observed based on GM colonization (Table S5). To summarize, a minority of differential features were specifically modulated by GM colonization or host genetic lineage, whereas the vast majority of features were modulated by a combination of the two factors. Furthermore, both individual metabolites and metabolic pathways were independently modulated based on genetic lineage or GM.

### Host genetic lineage influences bile acid metabolism

The divergent genetic lineages *Min*/J and *Min*/D had significantly altered adenoma susceptibility and metabolic profile. We therefore characterized genetic divergence between the *Min*/J and *Min*/D lines via ~30X whole-genome sequencing (WGS) on representative breeder female mice from each colony (see supplementary data and methods). SNPs and indels that were private to either *Min*/D or *Min*/J were categorized based on their predicted functional effect due to the nature of the variant using the Variant Effect Predictor (VEP) tool (Table S6). There were no private protein coding homozygous variants detected in either line, with all homozygous variants residing in noncoding regions. To interrogate overall effects of private mutations in each lineage, all private homozygous variants residing within or near known genes were used to identify over-represented KEGG [37] and REACTOME [38] biological pathways using the over-representation tool in InnateDB, which revealed over-representation of bile-acid metabolism in the *Min/*D line (Table S8) [39]. Variants near or within candidate genes contributing to bile acid metabolism included *Cyp39a1*, which codes for an enzyme involved in bile acid biosynthesis, and the intestinal bile acid transporter coded for by *Fabp6* [40, 41]. The *Min/*D line carried a single base (A) deletion in intron 1 of *Cyp39a1* at position chr17:43,674,583. *Min/*D also carried a T_6_ bp insertion within a poly T in the area upstream of *Fabp6* within the area of chr11:43,604,913-43,604,928. Notably, homozygous variants private to the *Min*/J line were detected near candidate genes *Myc* and *Dlg3* among other cancer related genes (Table S7).

### The *Min*/D *Fabp6* variant is associated with SI adenoma susceptibility

**Fig. 4:**
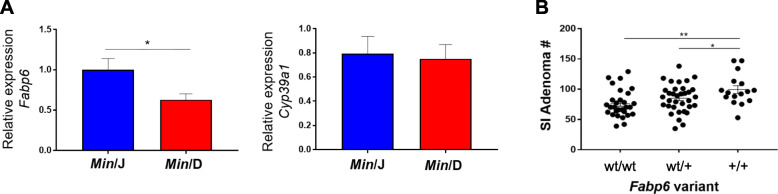
*Min*/D *Fabp6* variant association with SI adenoma susceptibility. **a** RT-qPCR comparison of relative expression of candidate genes *Fabp6* and *Cyp39a1* between *Min*/J and *Min*/D lineages, using ileal mucosal scrapes from normal intestinal epithelium and liver, respectively (*Min*/J, *n* = 10; *Min*/D, *n* = 12). **b** Scatter plots comparing mean (± SEM) SI tumor counts of N2 mice based on their status for the *Fabp6* insertion (wt/wt homozygous for absence of insertion, *n* = 29; wt/+ heterozygous for insertion, *n* = 34) +/+ homozygous for presence of the *Min/*D insertion, *n* = 16). *p < 0.05, **p < 0.01, ***p < 0.001; Student’s t-test (**a**) and ANOVA with the Student Newman-Keuls method (**b**)

Our WGS findings of variants associated with bile acid metabolism were particularly notable as they provide a possible explanation for the previously described changes in bile acid metabolites (Fig. 3d). However, it is unclear whether there are any functional consequences of the observed germline mutations. To determine whether detected *Fabp6* and *Cyp39a1* variants had potential downstream effects in the tissues they are normally expressed, we compared gene expression levels in the normal ileal epithelium and liver, respectively, of *Min*/J and *Min*/D mice. We found that *Min*/D mice had a significant reduction in *Fabp6* expression in the ileal epithelium compared to *Min*/J mice, while there were no differences in *Cyp39a1* mRNA levels in the liver (Fig. 4b).

We validated the *Fabp6* variant detected by WGS and further interrogated whether *Fabp6* is a possible modifier of adenoma susceptibility. The *Min*/D and *Min*/J parental lines were used to generate N2 mice. Tumor number assessment that was performed blinded for genotype showed a significant correlation with the *Fabp6* variants, where mice that were homozygous for the *Min*/D variant had the highest adenoma susceptibility. Those that were heterozygous displayed an intermediate phenotype, while mice that were homozygous for the *Min*/J variant had the lowest adenoma multiplicity (Fig. 4b). Thus, we observed differential *Fabp6* expression between the *Min*/J and *Min*/D lineages associated with the validated upstream insertion in *Min*/D mice, and further associated this variant with SI adenoma susceptibility in N2 mice.

### Colonic adenoma susceptibility is associated with changes in bile acid metabolism

**Fig. 5:**
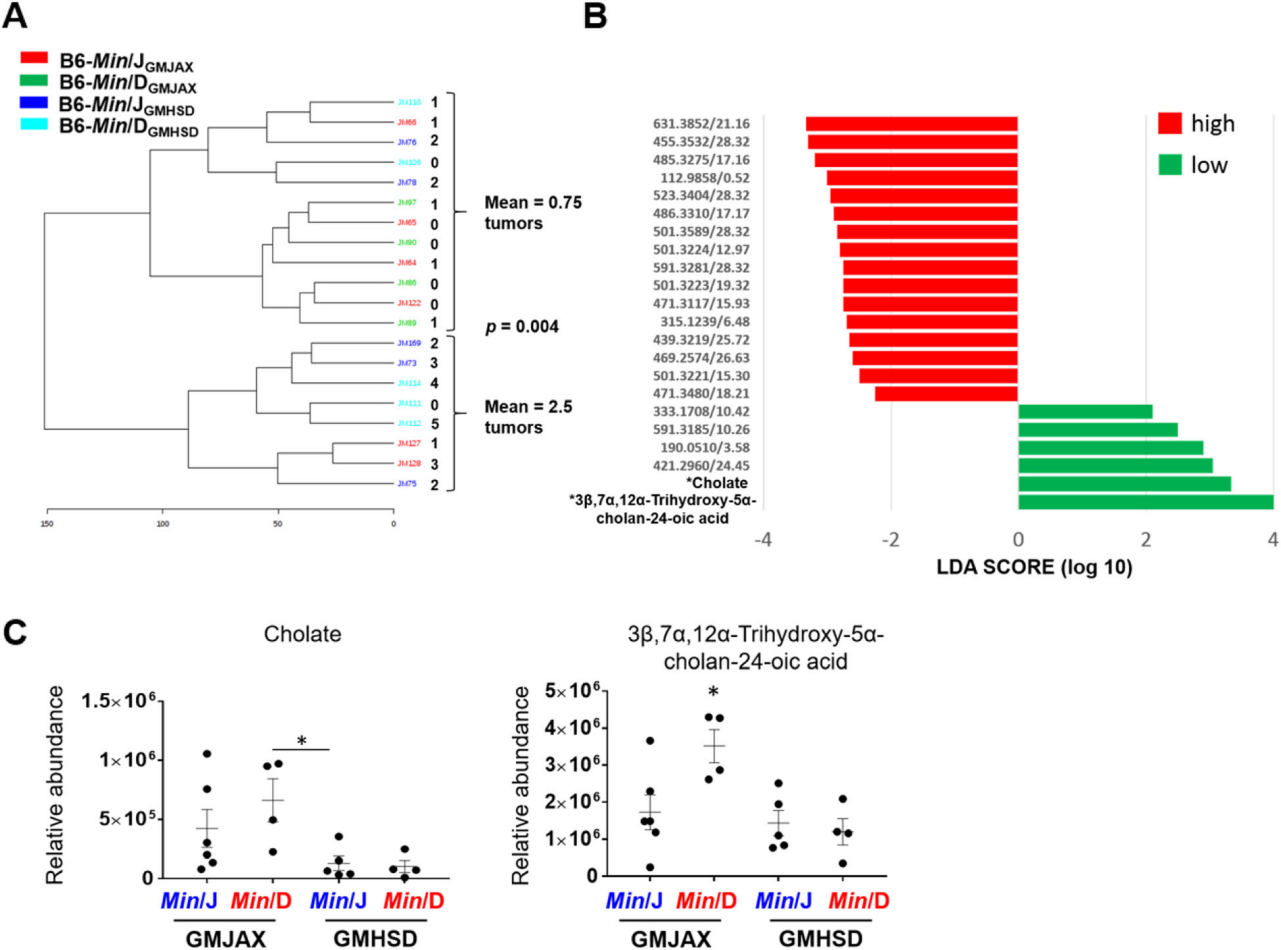
Colonic adenoma susceptibility is associated with changes in bile acid metabolism. **a** Dendrogram was generated based on the putative fecal metabolite features using the Euclidean distance of measurement and Wards clustering algorithm. The major root of the tree clustered samples independent of genetic lineage and GM profile. **b** Linear Discriminant Analysis (LDA) was used to identify the metabolites driving separation between the low-adenoma and high-adenoma clusters identified by the dendogram. **c** Scatter plots displaying relative abundance of two bile acids identified by tandem MS, significantly over-represented in the low-colonic adenoma group defined by the dendogram analysis (*Min*/J_GMJAX,_*n* = 6; *Min*/D_GMJAX,_*n* = 4; *Min*/J_GMHSD,_*n* = 5; *Min*/D_GMHSD_, *n* = 4). *p < 0.05, **p < 0.01; student’s t-test

We finally aimed to determine whether the fecal metabolome could account for the observed differences in colonic adenoma number between the original *Min*/D and *Min*/J colonies (Fig. 1a). An unsupervised dendrogram was generated to cluster the fecal metabolomes from 3 month old mice based on detected putative fecal metabolite features. The major root of the tree clustered samples into two distinct groups independent of genetic lineage and GM profile (Fig. 5a). Analysis of these two groups revealed that they correlated with colonic adenoma multiplicity, indicated by the numbers adjacent to the dendrogram, where one group had 0.75 ± 0.22 colon adenomas, while the other had 2.5 ± 0.57 colon adenomas. Linear Discriminant Analysis (LDA) was used to identify the metabolites driving separation between the low-adenoma and high-adenoma clusters. In total, we found 16 metabolites associated with the high-adenoma cluster, and 6 metabolites associated with the low-adenoma cluster (Fig. 5b). Of these metabolites, tandem MS enabled identification of two metabolites over-represented in the low-adenoma cluster, both of which were bile acid or bile acid derivatives. The relative abundance of putative cholate was primarily modulated by GM, while the abundance of putative 3β,7α,12α-Trihydroxy-5α-cholan-24-oic acid was dependent on both GM and genetic lineage (Fig. 5c). In conclusion, an unbiased clustering analysis of the fecal metabolomes of the rederived *Apc*^*Min*^ groups generated two primary groups, which were associated with colonic adenoma numbers. Identification of two of these metabolites driving the low- and high-adenoma clusters revealed elevated levels of two bile acid compounds in the low-adenoma group, while the remainder are currently uncharacterized.

## Discussion

The Min mouse is the single most cited mouse model of human cancer for nearly three decades, yielding an extraordinary wealth of information about the pathogenesis and treatment of human disease. However, the use of the Min mouse model for quantitative analysis of tumor susceptibility and treatment has been confounded by phenotypic variability of unknown origin, particularly with respect to adenoma multiplicity (Table 1). Here, we demonstrate how leveraging the observed phenotypic variability between Min colonies allows us to unravel the complex factors comprising disease susceptibility, with special focus on how host genetics and the gut microbiota (GM) collectively influence adenoma initiation. We utilized a multi-omics approach to integrate microbial community and host genomic data, and include the fecal metabolome to incorporate these data sets to provide new insight into the functional contributions of these interactions in CRC susceptibility.

We exploited our observation of a variable phenotype between two colonies that diverged from a common population; the original C57BL/6-*Apc*^*Min*^ colony generated and housed at the McCardle Laboratory at the University of Wisconsin (*Min*/D) and mice received from The Jackson Laboratory (*Min*/J) (Fig. 1a). Given the multi-generation segregation of the two colonies and the differences in selective pressures, we hypothesized that host genetic divergence would account for differences in adenoma susceptibility, despite having the same original inbred genetic background. Previous studies have also demonstrated that mice housed in different institutions have distinct GMs due to environmental differences, so we further hypothesized that the *Min*/D and *Min*/J colonies’ disparate GM communities could contribute to distinct phenotypes [22]. The original *Min*/D colony was rederived onto Hsd:CD1 surrogate dams from Envigo (previously Harlan) at the McCardle Laboratory, and therefore had a GM representing Envigo, while *Min*/J mice have a relatively less complex GM from The Jackson Laboratory [26]. To segregate the effects of host genetics and GM on adenoma susceptibility, we used a unique targeted rederivation approach in which we generated mice of the *Min*/D and *Min*/J genetic lineages each with a GM representing either Envigo (GMHSD) or The Jackson Laboratory (GMJAX) (Fig. 1b). Remarkably, we demonstrated that both genetic lineage and GM considerably influenced adenoma numbers. While *Min*/J mice colonized with GMHSD had increased adenoma numbers compared to our original *Min*/J colony, colonization of *Min*/D mice with GMJAX repressed adenoma numbers compared to the original *Min*/D colony, emphasizing a critical role for the GM in disease susceptibility in *Apc*^*Min*^ mice (Fig. 1c). Furthermore, rederived mice of the *Min*/D lineage developed more adenomas than their *Min*/J counterparts independent of GM colonization, indicating that genetic lineage similarly accounts for significant phenotypic variability (Fig. 1c). Thus, we demonstrate here that host genetics and the GM collectively accounted for the adenoma number disparity between two divergent colonies, additively determining adenoma multiplicity.

Microbial profiling via NGS of the 16S rRNA gene allows characterization of the GMJAX and GMHSD communities to identify OTUs associated with a protective versus deleterious phenotype. Analysis of β-diversity of the microbial taxa of GMHSD and GMJAX in the ileum and feces across multiple time points confirmed that these GMs are stably distinct from one another throughout the GI tract (Figs. 2 and S2). *Desulfovibrio* sp. and *Bilophila* sp., deltaproteobacteria producers of hydrogen sulfide (H_2_S) via sulfate and sulfite reduction, respectively, were 2–3 orders of magnitude higher in GMHSD compared to GMJAX in both ileal scrapes and feces (Tables S3 and S4) suggesting a potentially important role for sulfidogenic bacteria. A number of studies describe associations between H_2_S and CRC risk, indicating both pro- and anti-carcinogenic roles depending on concentration and route of cellular exposure [13, 42–44]. Of further interest, *Bilophila* sp., named for their close association with bile, is the only bacterial genera known to utilize taurine from taurine-conjugated bile acids for anaerobic respiration and H_2_S production [8, 45]. Due to its use of bile acids as a source of respiration, *B. wadsworthia* expands dramatically in western diets with higher fat content associated with increased taurine-conjugated bile acids, and thus presents a critical link between western diets, bile acid levels, sulfide production, and CRC risk [46]. While these suggestive results remain correlative, experiments focused on supplementing these bacteria in an environmentally controlled setting could provide additional insight into complex community structures and their role in CRC pathogenesis.

The emergence of targeted and untargeted metabolomics provide an avenue to interrogate metabolic changes in disease. While the high sensitivity of an untargeted approach yields large numbers of metabolites of interest, distinguishing these compounds from unclassified fragments or adducts poses a significant challenge [16]. This study in particular exemplifies the challenges of an untargeted approach, as the vast majority of detected differential metabolite features remain unidentified. It is also important to consider that more extensive annotation of certain metabolite classes may cause inherent bias when interpreting results. Thus, continued efforts to improve metabolite libraries, as well as bioinformatics pipelines that enable more efficient compound identification, are critical to the development of these approaches.

Despite these challenges, a wealth of information can be gleaned from controlled metabolomics studies. These data show that both the GM and host genetics shape the fecal metabolome, and in the process, could alter predisposition to adenoma initiation (Fig. 2a-b). Additional analysis enables mapping of differential putative compounds to metabolic pathways, and thus shows the perturbation of such metabolic pathways associated with pathology of interest. We identified dysregulation of bile acid metabolism in mice from the *Min*/D genetic lineage. The enterohepatic BA system is a classic example of the inter-dependent nature of host genetics and the GM. Host gene expression of enzymes responsible for primary BA biosynthesis, as well as intestinal transporters that recycle these BAs are required for functional enterohepatic circulation [47], while the GM de-conjugates and transforms primary bile acids as they traverse the GI tract to produce secondary BAs [48]. Thus, intra-intestinal levels of BAs depend upon cooperative genomic and microbial function.

Gleaning functional genomic significance of WGS variants is often especially challenging due to high numbers of misreads and unknown intergenic effects of poorly annotated functional elements. Thus, we used our metabolomics data, specifically identification of bile acid dysregulation, as a functional genomic tool to focus our variants of interest. We identified an insertion at a Spi1 transcription factor binding site of *Fatty acid binding protein 6* (*Fabp6*), a protein responsible for the re-uptake of bile acids in the ileum for enterohepatic recirculation [49], in *Min*/D mice. This variant was associated with decreased expression of *Fabp6* in the *Min*/D population, suggesting a functional role for the insertion. Previous studies have implicated *Fabp6* in human CRC where it was over-expressed in cancerous tissue relative to normal tissue. Counterintuitively, higher expression levels of *Fabp6* within tumors correlated with smaller tumors and less metastasis, suggesting its potential role in early carcinogenesis [41]. Decreased expression of *Fabp6* in normal ileal epithelium associated with increased adenomagenesis in *Min*/D mice, as well as a significant association between the *Fabp6* variant and SI adenoma multiplicity in N2 mice, support the proposed role for *Fabp6* in tumor initiation (Fig. 4b).

While it is difficult to discern contributing factors to colonic adenoma development in *Apc*^*Min*^ mice due to low colon tumor numbers and an incompletely penetrant phenotype, metabolomics may provide a foundation for identifying changes associated with a more severe or suppressed colonic phenotype. An unbiased analysis of fecal metabolites in our rederived groups separated the metabolic profiles into two distinct groups defined by colonic tumor number. Among several associated compounds, we identified two bile acids where increased abundance was associated with the low-adenoma group (Fig. 5). Previous studies have implicated secondary bile acids such as deoxycholic acid (DCA) in CRC pathogenesis through mechanisms including oxidative damage and mitochondrial dysfunction, while primary bile acids can inhibit adenoma formation [50–52]. Cholic acid (CA) is a primary bile acid converted to DCA by the gut microbiota [53]. Enrichment of cholate in the low-adenoma group, and its association with GMJAX-colonized mice, may indicate that GMJAX converts CA to DCA less efficiently than GMHSD, and therefore confers a suppressed adenoma phenotype. These results highlight the diversity of bile acids and their potential effects on host cell proliferation in CRC, and suggest that carcinogenesis may depend upon a delicate balance between the two. However, further targeted studies are required to better characterize dysregulation of primary and secondary bile acids, and to determine how genetic variants and the microbiota each influence these metabolites.

## Conclusions

Colorectal cancer is a classic example of a multifaceted disease with complex biological systems contributing to overall susceptibility and pathogenesis. Here, we demonstrate that complex GM communities and host genetics both independently and additively modulate adenoma development in *Apc*^*Min*^ mice. We utilized a metabolomics platform to show that genetically divergent host genomes and complex GM interactively shape the intestinal metabolome. Our strategy of utilizing untargeted metabolomics data as a functional genomics tool enabled us to focus our attention to WGS variants of consequence. Thus, we demonstrate a tactic to extract pathologically relevant functional candidate variants from large sequencing data sets. This work provides a platform for both mechanistic links between genetic variants and the GM as well as biomarker discovery. Furthermore, this data provides a clear explanation for much of the variability observed in the *Apc*^*Min*^ tumor phenotype throughout its use over the course of several decades, and may explain substantial differences in susceptibility to CRC across different human populations. Finally, this approach was relatively non-invasive and can be translated to human studies, integrating the complicated interactive nature of the host genome, the GM, and the metabolome to create individualized risk assessment and tailored preventive medicine strategies.

## Supplementary information

**Additional file 1.**

**Additional file 2.**

**Additional file 3.**

**Additional file 4.**

## Data Availability

The datasets in this publication have been made available for public access. Microbiome sequence data is available in the NCBI Sequence Read Archive. *SRA****:*****SRP216253***BioProject:* PRJNA555614. The 16S sample metadata is listed in the supplementary data. The metabolomics metadata is listed in supplemental and data is available through the public database metabolomics workbench (amoslandgrafj_20200522_151621_mwtab).

## Abbreviations

CRC: colorectal cancer
GM: gut microbiota
Apc: Adenomatous polyposis coli
Min: Multiple intestinal neoplasia allele
*Min*/J: C57BL/6 J-*Apc*^Min^ mice
*Min/*D: C57BL/6JD-*Apc*^Min^ mice
*Fabp6*: Fatty acid binding protein 6
BA: bile acids
H_2_S: hydrogen sulfide
GMJAX: complex gut microbiota with original origins from the Jackson Laboratory
GMHSD: complex gut microbiota with original origins from Harlan Sprague Dawley (Envigo)
