# Supplementary material for: Integration of genomics, metagenomics, and metabolomics to identify interplay between susceptibility alleles and microbiota in adenoma initiation
Source: BMC Cancer. 2020 Jun 29;20:600. doi: 10.1186/s12885-020-07007-9 (PMC7322931; doi:10.1186/s12885-020-07007-9)
Supplement: Supplementary file 4 — Additional file 4. [file 12885_2020_7007_MOESM4_ESM.docx]

**SUPPLEMENTARY MATERIALS**

**SUPPLEMENTARY FIGURE LEGENDS**

**Figure S1. Effects of genetic lineage and GM on adenoma susceptibility by sex.** Scatter plots comparing mean (± SEM) small intestinal (SI) and colon adenoma counts of the original B6-*Apc^Min^* colony generated at UW McArdle Laboratory (*Min*/D) to B6-*Apc^Min^* mice acquired from The Jackson Laboratory and maintained at University of Missouri (*Min*/J) of females **(A)** (*Min*/D, n = 31; *Min*/J, n = 3), and males **(B)** (*Min*/D, n = 34; *Min*/J, n = 19). Scatter plots comparing mean (± SEM) SI and colon adenoma counts of the four rederived groups, including each genetic lineage (*Min*/J and *Min*/D) rederived with two complex GMs of females **(C)** (*Min*/J_GMJAX,_ *n* = 4; *Min*/D_GMJAX,_ *n* = 9; *Min*/J_GMHSD,_ *n* = 10; *Min*/D_GMHSD_, *n* = 4) and males **(D)** (*Min*/J_GMJAX,_ *n* = 9; *Min*/D_GMJAX,_ *n* = 9; *Min*/J_GMHSD,_ *n* = 9; *Min*/D_GMHSD_, *n* = 6). **p*<0.05, ***p*<0.01, ****p*<0.001; student’s t-test and Two-way ANOVA with the Student Newman-Keuls method.

**Figure S2. Analysis of α- and β-diversity measures of GMJAX and GMHSD. A,** Unweighted PCoA representing differences in β-diversity at the Operational Taxanomic Unit (OTU) level between complex GM profiles of CMTR offspring in feces at 3 months of age and **B,** unweighted PCoA of 1 month fecal OTUs separated by sex. **C,** Differences in GM richness (Chao1 index) and α-diversity (Shannon Index) are shown with Tukey’s boxplots. **p*<0.05, ***p*<0.01, ****p*<0.001; Kruskal-Wallis ANOVA on ranks with Dunn’s Method for Multiple Comparisons.

**Figure S3. GM profile and genetic lineage modulate relative abundances of specific fecal metabolites. A,** Scatter plots showing relative abundance of fecal metabolites modulated by the GM independent of genetic lineage (GMJAX, n = 4; GMHSD, n = 5). **B,** Scatter plots showing relative abundance of fecal metabolites modulated by genetic lineage independent of GM (*Min*/J, n = 6; *Min*/D, n = 4). Metabolites are labeled by mass-to-charge ratio/retention time. ****p*<0.001.

**Figure S4. Sequencing analysis of detected *Fabp6* variant.** Chromatogram of Sanger sequencing results for validation of the 5-bp deletion detected at position 43604912 of chromosome 11 in *Min*/J WGS. DNA extracted from ear punches of representative *Min*/D (top) and *Min*/J (bottom) mice, PCR amplified for the region of interest, and TA-cloned using the pCR 4-TOPO vector. The T7 sequencing primer (5’-TAATACGACTCACTATAGGG-3’ was used for the sequencing reaction.

**SUPPLEMENTARY FIGURES**

**Figure S1**

**
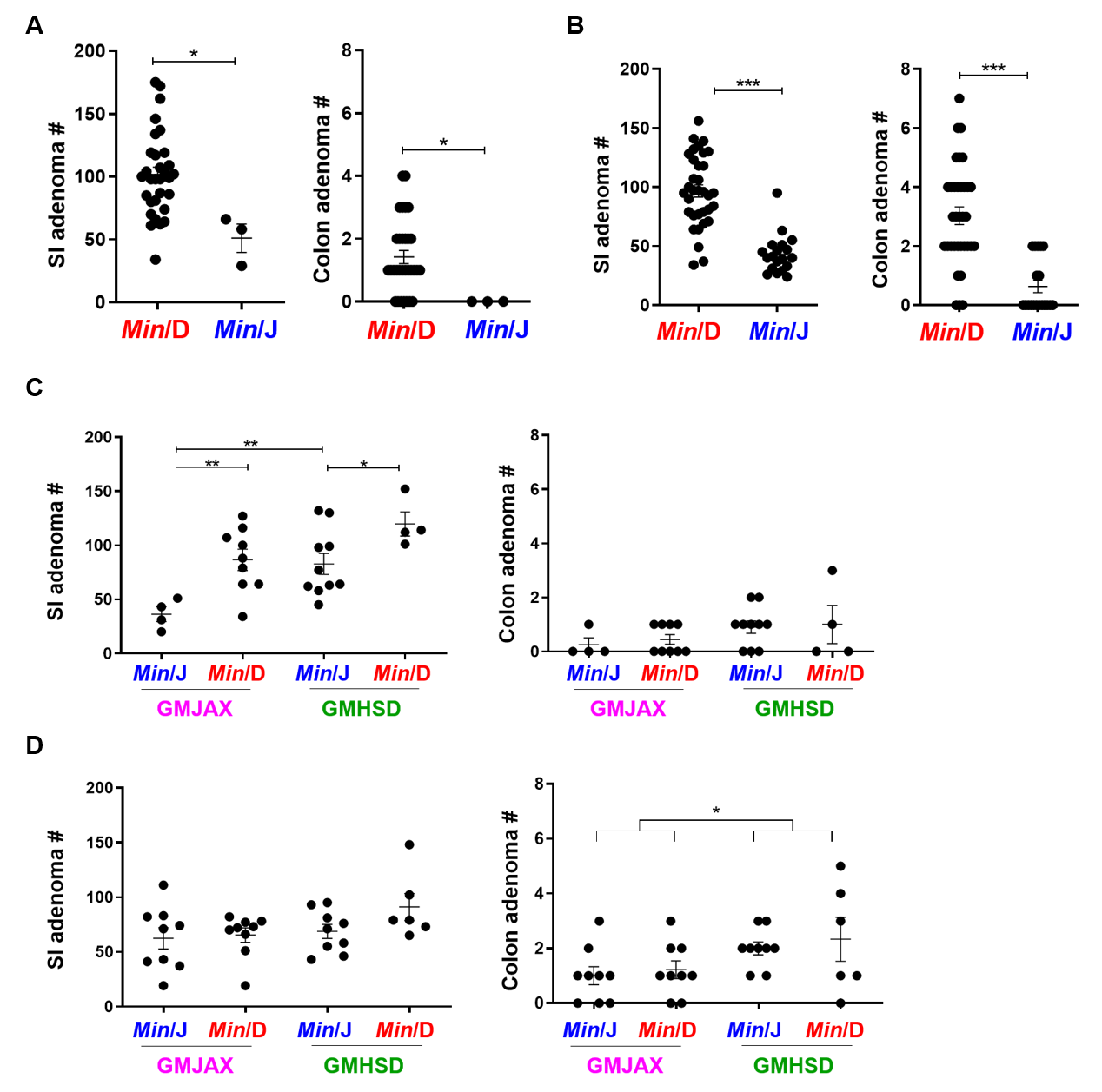
**

**Figure S2**


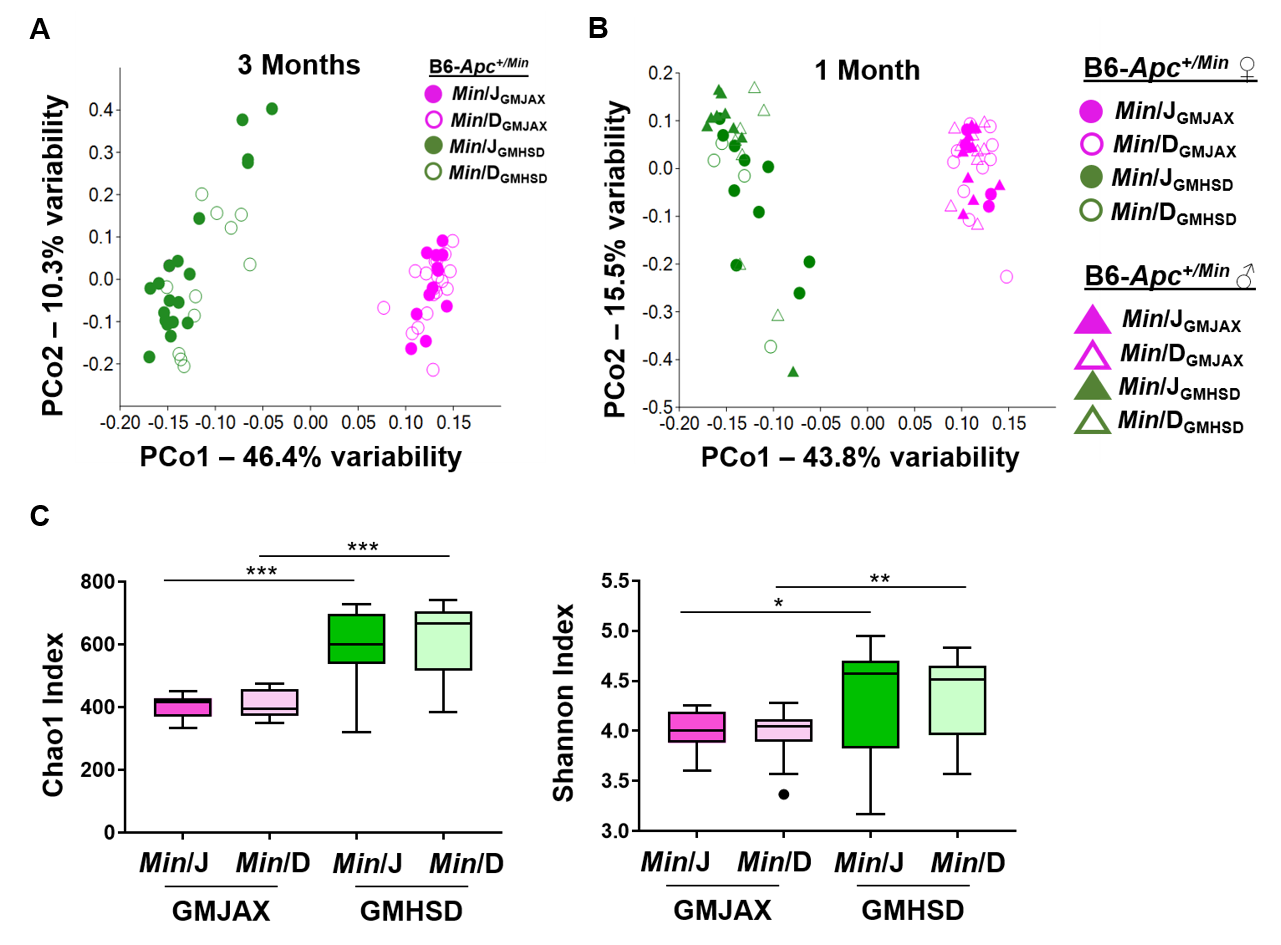


**Figure S3**


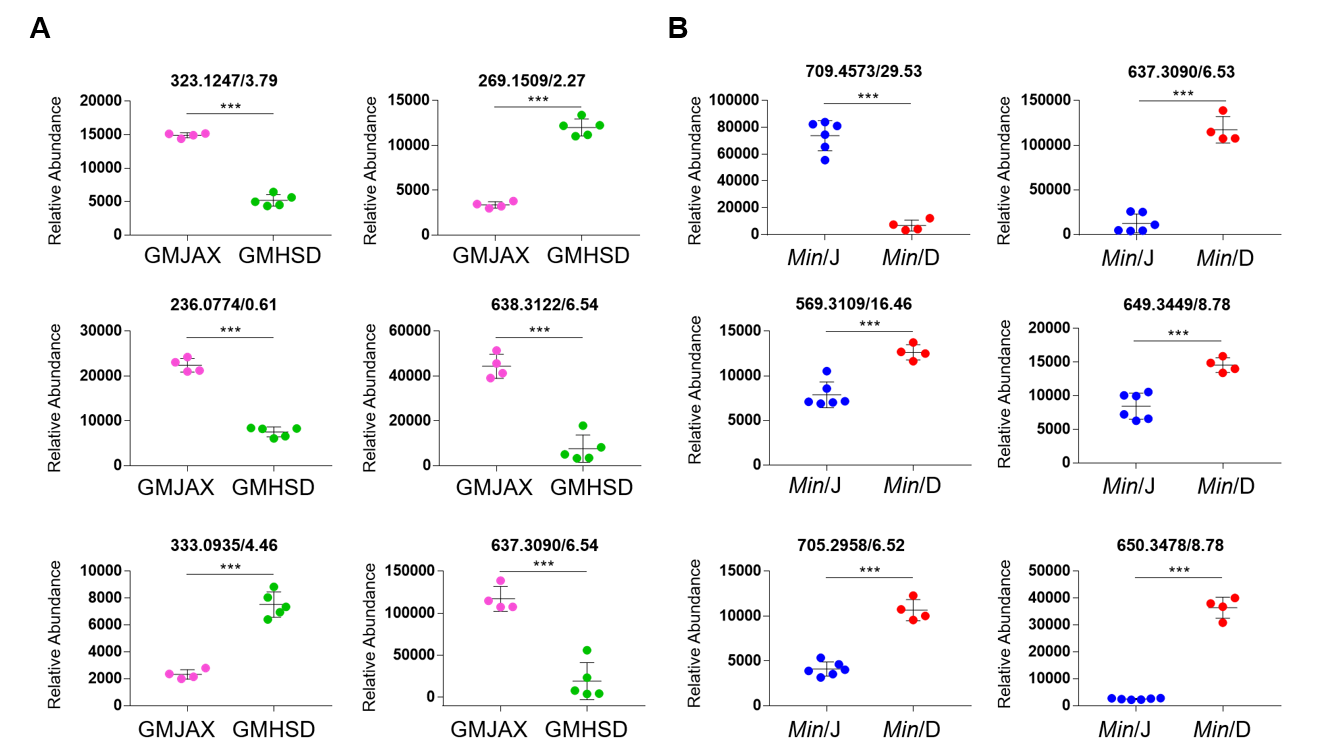


**Figure S4**


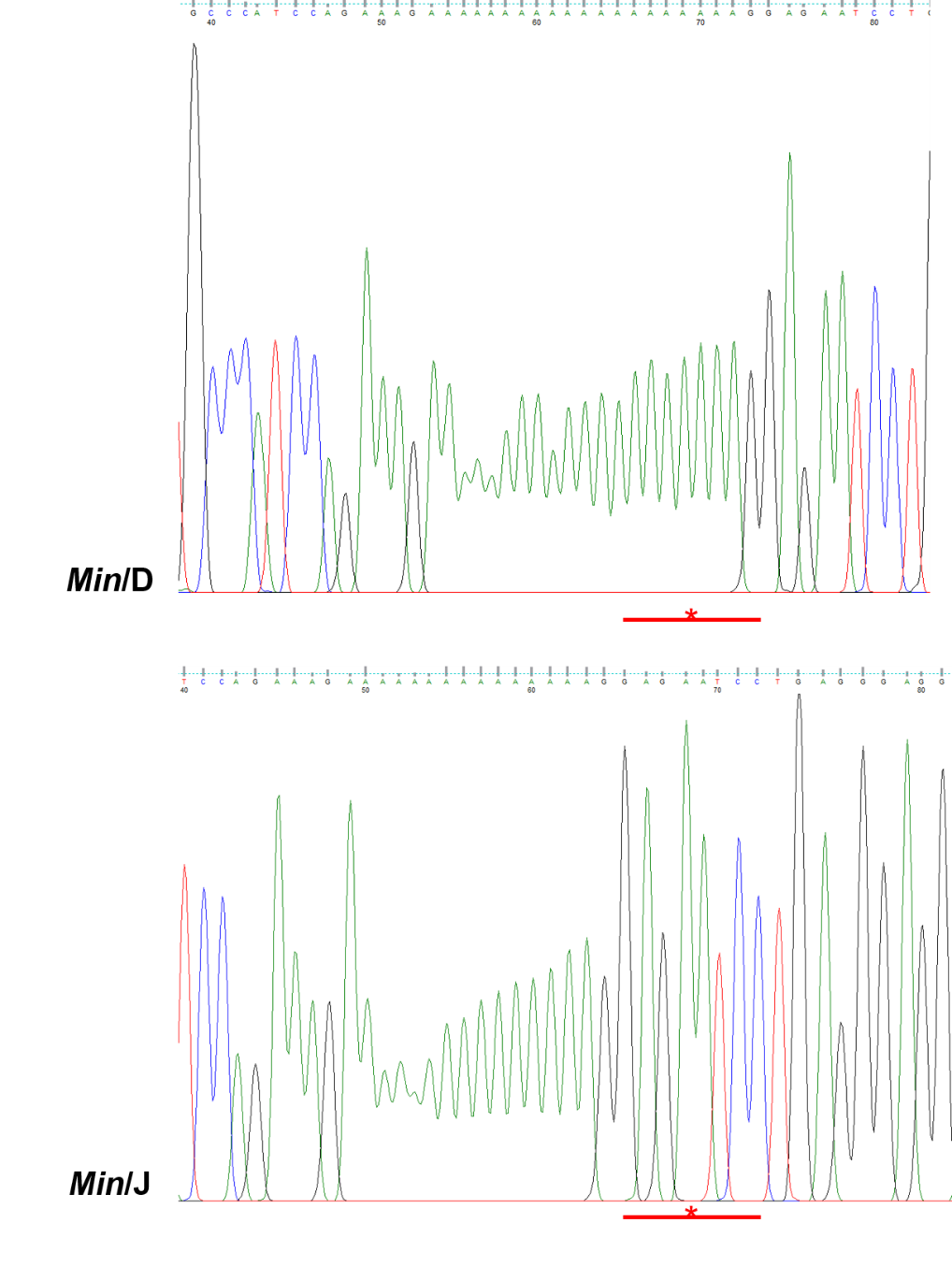


*Fabp6*

**SUPPLEMENTARY TABLES**

| **Table S1. Two-way PERMANOVA community analysis of fecal GM and host genetic effects on β-diversity** | | | | |
| --- | --- | --- | --- | --- |
|  | **Bray Curtis** | | **Jaccard** | |
| **Variable** | ***P* value** | ***F* value** | ***P* value** | ***F* value** |
| **GM (JAX vs HSD)** | 0.0001 | 13.68 | 0.0001 | 43.26 |
| **Genetics (*Min*/J vs *Min*/D** | .068 | 2.16 | 0.0025 | 4.36 |
| **Interaction** | 0.716 | -5.18 | 0.85 | -5.23 |

Bray Curtis and Jaccard’s dissimilarity indices, based on 9999 and *p* ≤ 0.05 is considered significant.

| **Table S2. Two-way PERMANOVA Community analysis of ileal GM and host genetic effects on β-diversity** | | | | |
| --- | --- | --- | --- | --- |
|  | **Bray Curtis** | | **Jaccard** | |
| **Variable** | ***P* value** | ***F* value** | ***P* value** | ***F* value** |
| **GM (JAX vs HSD)** | 0.0004 | 5.53 | 0.0001 | 9.46 |
| **Genetics (*Min*/J vs *Min*/D** | .268 | 1.06 | 0.0020 | 2.43 |
| **Interaction** | 0.758 | -5.36 | 0.378 | -4.18 |

Bray Curtis and Jaccard’s dissimilarity indices, based on 9999 and *p* ≤ 0.05 is considered significant.

| **Table S3. Significantly different fecal OTUs between GMJAX and GMHSD** | | | | | |
| --- | --- | --- | --- | --- | --- |
| **Significant OTUs in B6-*Apc^+/Min^* (*p* < 0.001)** | | **GMHSD**  **relative to GMJAX** | | **GMJAX** | **GMHSD** |
| **Phylum** | **OTU** | ***p-*value** | **Mean Fold Change** | **Mean Relative Abundance (±SEM)** | **Mean Relative Abundance (±SEM)** |
| *Proteobacteria* | Genus *Bilophila* | 2.18E-15 | 523.12 | 7.29E-07  ±5.14E-07 | 0.0025  ±0.000254109 |
| *Bacteroidetes* | Genus *Odoribacter* | 2.19E-15 | 685.34 | 2.95E-06  ±1.23E-06 | 0.0044  ±0.001223686 |
| *Firmicutes* | Family *Peptococcaceae* | 1.36E-14 | 0.16577 | 0.0096  ±0.0007 | 0.0016  ±0.00017436 |
| *Bacteroidetes* | Genus *Rikenella* | 3.31E-11 | 662.83 | 2.92E-06  ±1.36E-06 | 0.0043  ±0.000572708 |
| *Firmicutes* | Genus *Ruminococcaceae* NK4A214 | 4.95E-11 | 5.7893 | 0.0003  ±3.57E-05 | 0.0015  ±0.000161456 |
| *Firmicutes* | Genus *Lachnospiraceae* UCG-009 | 1.70E-10 | 0.045779 | 0.0006  ±6.484E-05 | 2.27E-05  ±1.23817E-05 |
| *Bacteroidetes* | Genus *Bacteroides* | 8.19E-10 | 0.26689 | 0.0918  ±0.0084 | 0.0245  ±0.002462137 |
| *Firmicutes* | Genus Family XIII AD3011 group | NA | NA | ND | 0.0001  ±1.95E-05 |
| *Bacteroidetes* | *Parabacteroides goldsteinii* | 2.46E-09 | 0.28362 | 0.0019  ±0.0002 | 0.0005  ±7.69066E-05 |
| *Firmicutes* | Genus *Ruminococcaceae* UCG-009 | 3.49E-09 | 218.38 | 4.00E-07  ±4.00E-07 | 0.0010  ±0.000154019 |
| *Proteobacteria* | Genus *Parasutterella* | 4.54E-09 | 0.002419 | 2.04E-06  ±8.68E-07 | 0.0024  ±0.000494444 |
| *Firmicutes* | Genus Family XIII UCG-001 | 8.86E-09 | 49.401 | 1.12E-06  ±6.32E-07 | 0.0002  ±3.93035E-05 |
| *Firmicutes* | Genus *Ruminococcaceae* UCG-014 | 2.84E-08 | 10.455 | 0.0017  ±0.00021 | 0.0182  ±0.002792152 |
| *Actinobacteria* | Genus *Asaccharobacter* | NA | NA | ND | 6.78E-05  ±1.16376E-05 |
| *Firmicutes* | Genus *Lachnospiraceae* UCG-010 | 1.09E-07 | 0.15101 | 0.0003  ±3.92E-05 | 4.43E-05  ±6.38491E-06 |
| *Firmicutes* | Genus *Ruminococcaceae* UCG-014 | 1.43E-07 | 364.11 | 3.68E-06  ±1.34E-06 | 0.0026  ±0.000467732 |
| *Firmicutes* | Family *Peptococcaceae* | 1.59E-07 | 189.63 | 1.79E-06  ±9.52E-07 | 0.0010  ±0.000191244 |
| *Bacteroidetes* | Genus *Fluviicola* | NA | NA | ND | 0.0001  ±2.15285E-05 |
| *Bacteroidetes* | Genus *Rikenellaceae* RC9 | 4.87E-07 | 1801.1 | 1.47E-06  ±7.21E-07 | 0.0094  ±0.001807182 |
| *Bacteroidetes* | *Alistipes* CC-5826 | 5.53E-07 | 30.486 | 3.51E-07  ±3.51E-07 | 0.0001  ±2.55578E-05 |
| *Firmicutes* | Genus *Ruminococcaceae* UCG-003 | 7.59E-07 | 55.968 | 1.70E-05  ±4.49E-06 | 0.0011  ±0.000211306 |
| *Firmicutes* | Family *Clostridiales* vadinBB60 | NA | NA | ND | 4.79E-05  ±3.10416E-05 |
| *Firmicutes* | Genus *Ruminiclostridium* 5 | 1.02E-06 | 60.332 | 9.00E-06  ±2.49E-06 | 0.0007  ±0.000136352 |
| *Firmicutes* | Family *Erysipelotrichaceae* | 1.25E-06 | 8.3106 | 0.0001  ±3.36E-05 | 3.44E-05  ±1.11112E-05 |
| *Proteobacteria* | Genus *Oxalobacter* | 1.58E-06 | 0.043163 | 0.0001  ±1.83E-05 | 6.13E-07  ±6.13333E-07 |
| *Bacteroidetes* | Genus *Alloprevotella* | 1.63E-06 | 610.15 | 6.77E-06  ±1.45E-06 | 0.0056  ±0.001143238 |
| *Proteobacteria* | Genus *Parasutterella* | 1.80E-06 | 427.03 | 0.0090  ±0.0012 | 2.044E-05  ±3.65132E-06 |
| *Firmicutes* | Family *Christensenellaceae* | 3.38E-06 | 0.28271 | 0.0003  ±4.07E-05 | 9.68E-05  ±2.30679E-05 |
| *Actinobacteria* | Genus *Senegalimassilia* | NA | NA | ND | 1.41E-05  ±2.80013E-06 |
| *Firmicutes* | Genus *Christensenellaceae* R-7 | 5.09E-06 | 33.441 | 8.10E-07  ±4.56E-07 | 0.0002  ±3.30134E-05 |
| *Tenericutes* | Genus *Anaeroplasma* | 5.77E-06 | 0.028716 | 0.0165  ±0.0030 | 0.0005  ±0.000202858 |
| *Firmicutes* | Genus *Ruminiclostridium* 5 | 7.04E-06 | 0.13776 | 0.0120  ±0.0020 | 0.0017  ±0.00019513 |
| *Deferribacteres* | Genus *Mucispirillum* | 7.12E-06 | 1078 | 5.15E-06  ±1.52E-06 | 0.0088  ±0.001940521 |
| *Firmicutes* | Genus *Coprococcus* | 1.06E-05 | 0.34219 | 0.0054  ±0.0007 | 0.0018  ±0.000258107 |
| *Cyanobacteria* | Order *Gastranaerophilales* | 1.38E-05 | 725.14 | 2.32E-06  ±8.83E-07 | 0.0042  ±0.001972326 |
| *Proteobacteria* | Genus *Desulfovibrio* | 1.55E-05 | 1566.8 | 7.18E-06  ±1.89E-06 | 0.0154  ±0.003556409 |
| *Firmicutes* | Genus *Ruminococcaceae* UCG-013 | NA | NA | ND | 4.12E-05  ±9.24984E-06 |
| *Firmicutes* | Genus *Ruminococcaceae* UCG-005 | 2.45E-05 | 0.27122 | 0.0012  ±0.0002 | 0.0003  ±5.42308E-05 |
| *Firmicutes* | Genus *Erysipelatoclostridium* | 3.64E-05 | 38.155 | 8.46E-07  ±5.90E-07 | 0.0002  ±4.3734E-05 |
| *Firmicutes* | *Catabacter hongkongensis* | NA | NA | ND | 3.06E-05  ±7.15299E-06 |
| *Firmicutes* | Genus Family XIII AD3011 group | 4.15E-05 | 0.49768 | 0.0003  ±2.19E-05 | 0.0001  ±1.74932E-05 |
| *Firmicutes* | Genus *Ruminococcaceae* UCG-011 | 4.46E-05 | 313.95 | 3.12E-06  ±1.04E-06 | 0.0020  ±0.000496075 |
| *Tenericutes* | Order *Mollicutes* RF9.1 | NA | NA | ND | 0.0002  ±4.80153E-05 |
| *Firmicutes* | Genus *Anaerovorax* | 7.77E-05 | 0.33895 | 0.0001  ±1.46E-05 | 4.82E-05  ±1.76295E-05 |
| *Firmicutes* | Family *Christensenellaceae* | 0.0001 | 2.5038 | 2.75E-05  ±3.87E-06 | 7.02E-05  ±1.0496E-05 |
| *Firmicutes* | Family *Lachnospiraceae* | 0.000101 | 5.0579 | 0.0005  ±8.42E-05 | 0.0024  ±0.000490801 |
| *Firmicutes* | Genus *Ruminiclostridium* 5 | 0.00018 | 3.2504 | 7.05E-05  ±1.50E-05 | 0.0002  ±3.97143E-05 |
| *Bacteroidetes* | Genus *Prevotella* | 0.000218 | 0.46679 | 0.0067  ±0.0008 | 0.0031  ±0.000442018 |
| *Bacteroidetes* | Genus *Odoribacter*.1 | 0.000236 | 0.1121 | 0.0097  ±0.0008 | 0.0011  ±0.000171828 |
| *Firmicutes* | Family *Ruminococcaceae*.3 | 0.000283 | 186.66 | 2.64E-06  ±1.26E-06 | 0.0012  ±0.0003265 |
| *Firmicutes* | Family *Ruminococcaceae*.2 | NA | NA | ND | 1.54E-05  ±4.0868E-06 |
| *Firmicutes* | Family *Lachnospiraceae*.4 | NA | NA | ND | 1.86E-05  ±5.06681E-06 |
| *Firmicutes* | Family *Lachnospiraceae*.2 | 0.000373 | 415.54 | 1.59E-06  ±9.53E-07 | 0.0023  ±0.000648554 |
| *Bacteroidetes* | Genus *Bacteroides*.1 | 0.00038 | 0.21468 | 0.0001  ±1.97E-05 | 2.07E-05  ±7.15312E-06 |
| *Firmicutes* | Genus *Ruminiclostridium* | 0.000385 | 527.2 | 3.97E-07  ±3.97E-07 | 0.0024  ±0.000681413 |
| *Firmicutes* | Genus *Ruminococcaceae* UCG-005.1 | NA | NA | ND | 6.99E-05  ±1.99217E-05 |
| *Firmicutes* | bacterium NLAE-zl-H60 | 0.000733 | 0.15777 | 0.0018  ±0.0004 | 0.0003  ±7.82195E-05 |
| *Firmicutes* | Genus *Peptoclostridium* | 0.000814 | 0.061672 | 0.0014  ±0.0004 | 8.67E-05  ±3.85033E-05 |

ND: Relative abundance was below detectable limits by 16S sequencing

NA: Statistical comparison was not applicable due to a relative abundance below detectable limits (ND) in at least one GM group

| **Table S4. Significantly different ileal scrape OTUs between GMJAX and GMHSD** | | | | | |
| --- | --- | --- | --- | --- | --- |
| **Significant OTUs in B6-*Apc^+/Min^* (*p* < 0.001)** | | **GMHSD**  **relative to GMJAX** | | **GMJAX** | **GMHSD** |
| **Phylum** | **OTU** | ***p-*value** | **Mean Fold Change** | **Mean Relative Abundance (±SEM)** | **Mean Relative Abundance (±SEM)** |
| *Proteobacteria* | Genus *Parasutterella* | 4.56E-31 | 481.21 | 9.63E-06  ±6.99E-06 | 0.0101  ±0.0012 |
| *Proteobacteria* | Genus *Desulfovibrio* | 1.64E-15 | 201.56 | 5.72E-05  ±5.72E-05 | 0.0140  ±0.0028 |
| *Bacteroidetes* | Family *Muribaculaceae* | 4.02E-15 | 47.486 | 0.0001  ±5.64E-05 | 0.0072  ±0.0010 |
| *Firmicutes* | Genus *Butyricicoccus* | NA | NA | ND | 0.0009  ±0.0003 |
| *Firmicutes* | Family *Peptostreptococcaceae* | 5.92E-10 | 0.036848 | 0.0148  ±0.0042 | 0.0005  ±0.0002 |
| *Cyanobacteria* | Order *Gastranaerophilales.1* | NA | NA | ND | 0.0031  ±0.0013 |
| *Proteobacteria* | Genus *Bilophila* | NA | NA | ND | 0.0014  ±0.0003 |
| *Firmicutes* | Genus *Lachnospiraceae UCG-001* | NA | NA | ND | 0.0017  ±0.0009 |
| *Firmicutes* | Genus *Ruminococcaceae UCG-014.1* | 2.34E-09 | 37.965 | 0.0003  ±0.0001 | 0.0113  ±0.0029 |
| *Bacteroidetes* | Genus *Muribaculum* | 1.90E-08 | 0.41829 | 0.0565  ±0.0048 | 0.0236  ±0.0024 |
| *Bacteroidetes* | Genus *Alloprevotella.2* | 1.32E-07 | 22.386 | 5.95E-05  ±5.32E-05 | 0.0016  ±0.0004 |
| *Bacteroidetes* | Genus *Rikenella* | NA | NA | ND | 0.0007  ±0.0002 |
| *Firmicutes* | Genus *Ruminococcaceae UCG-014* | 6.87E-07 | 13.226 | 0.0001  ±4.89E-05 | 0.0015  ±0.00041 |
| *Actinobacteria* | Genus *Bifidobacterium* | 1.19E-06 | 29.214 | 8.98E-05  ±5.08E-05 | 0.0029  ±0.0008 |
| *Bacteroidetes* | Genus *Alistipes.1* | 2.19E-06 | 7.0451 | 0.0008  ±0.00054 | 0.0054  ±0.0019 |
| *Verrucomicrobia* | Genus *Akkermansia* | 2.71E-06 | 27.871 | 0.0011  ±0.0003 | 0.0301  ±0.0064 |
| *Tenericutes* | Genus *Anaeroplasma* | 6.98E-06 | 0.12163 | 2.13E-05  ±1.49E-05 | 0.0118  ±0.0093 |
| *Firmicutes* | Genus *Eisenbergiella* | NA | NA | ND | 0.0013  ±0.0010 |
| *Proteobacteria* | Genus *Parasutterella.1* | 1.50E-05 | 0.22455 | 0.0218  ±0.0035 | 0.0049  ±0.0011 |
| *Firmicutes* | Genus *Eubacterium oxidoreducens* | 2.62E-05 | 0.076388 | 0.0054  ±0.0011 | 0.0004  ±0.0001 |
| *Firmicutes* | Genus *Dubosiella* | 3.69E-05 | 58.775 | 6.35E-06  ±6.35E-06 | 0.0011  ±0.0003 |
| *Tenericutes* | Genus *Anaeroplasma.1* | 5.69E-05 | 360.57 | 0.02070  ±0.0049 | 0.0025  ±0.0013 |
| *Deferribacteres* | Genus *Mucispirillum* | 9.35E-05 | 89.705 | 4.75E-05  ±2.62E-05 | 0.0052  ±0.0026 |
| *Firmicutes* | Genus *Roseburia.1* | NA | NA | ND | 0.0004  ±0.0001 |
| *Firmicutes* | Genus *Ruminococcaceae UCG-003* | 0.000125 | 0.18607 | 0.0045  ±0.0008 | 0.0008  ±0.0002 |
| *Firmicutes* | Genus *UBA1819* | NA | NA | ND | 0.00015659  ±4.86E-05 |
| *Proteobacteria* | *Azospirillum sp. 47_25* | NA | NA | ND | 0.001290673  ±0.0006 |
| *Firmicutes* | Genus *Butyricicoccus.1* | 0.000269 | 0.25529 | 0.0010  ±0.0002 | 0.000249552  ±7.35E-05 |
| *Firmicutes* | Genus *Lachnospiraceae UCG-006* | 0.000665 | 3.2945 | 0.0009  ±0.0003 | 0.0030  ±0.0008 |
| *Cyanobacteria* | Order *Gastranaerophilales* | 0.000736 | 7.421 | 5.37E-05  ±3.93E-05 | 0.0004  ±0.0002 |
| *Firmicutes* | Genus *Coprococcus* | 0.000789 | 145.18 | 8.54E-06  ±8.54E-06 | 0.0030  ±0.0022 |
| *Firmicutes* | Genus *Erysipelatoclostridium* | NA | NA | ND | 0.0005  ±0.0002 |
| *Firmicutes* | Genus *Roseburia* | 0.000911 | 0.25049 | 0.0035  ±0.0012 | 0.0009  ±0.0003 |
| *Firmicutes* | Genus *Tyzzerella* | NA | NA | ND | 4.56E-05  ±3.41E-05 |

ND: Relative abundance was below detectable limits by 16S sequencing

NA: Statistical comparison was not applicable due to a relative abundance below detectable limits (ND) in at least one GM group

| **Table S5. Summary of metabolic pathways modulated by genetic lineage and GM** | | | |
| --- | --- | --- | --- |
| **Pathway Modulated** | **Modulating factor** | ***p* value (corrected)** | **Overlapping metabolites** |
| Sphingosine/sphingosine-1-phosphate metabolism | GM | 3.3 x 10^-6^ | 3 |
| Morphine biosynthesis | GM | 1.7 x 10^-5^ | 7 |
| Bile acid biosynthesis/metabolism | Genetic lineage | 0.1 | 4 |

| **Table S6. Summary of heterozygous and homozygous private variants associated with *Min*/J and *Min*/D colonies** | | | | | | | | |
| --- | --- | --- | --- | --- | --- | --- | --- | --- |
|  | **VEP 0** | | **VEP 1** | | **VEP 2** | | **Total** | |
|  | **Het** | **Hom** | **Het** | **Hom** | **Het** | **Hom** | **Het** | **Hom** |
| ***Min*/D** | 14,996 | 475 | 3,917 | 109 | 54 | 0 | 18,967 | 584 |
| ***Min*/J** | 18,467 | 388 | 4,772 | 115 | 94 | 1 | 23,333 | 504 |

| **Table S7. Significantly over-represented biological pathways and candidate genes identified in *Min*/J** | | | |
| --- | --- | --- | --- |
| **Pathway name** | **Database** | ***P* value (corrected)** | **Candidate genes** |
| Neurotransmitter Receptor Binding | REACTOME | 1.29 x 10^-2^ | *Dlg3, Gabrb1, Gria1* |
| Ion channel transport | REACTOME | 1.53 x 10^-2^ | *Casq2, Gabrb1, Mcoln3* |
| Neuronal System | REACTOME | 1.57 x 10^-2^ | *Dlg3, Gabrb1, Gria1, Kcnb2* |
| Transmission across Chemical Synapses | REACTOME | 1.75 x 10^-2^ | *Dlg3, Gabrb1, Gria1* |
| Stimuli-sensing channels | REACTOME | 3.52 x 10^-2^ | *Casq2, Mcoln3* |
| Small cell lung cancer | KEGG | 3.84 x 10^-2^ | *Fhit, Myc* |
| TGF-beta signaling pathway | KEGG | 4.21 x 10^-2^ | *Acvr1, Myc* |
| Ubiquitin mediated proteolysis | KEGG | 6.97 x 10^-2^ | *Herc2, Ube2e3* |

Candidate genes are genes that are found in the over-represented pathways and contained SNPs or indels private to the

*Min*/J line. Corrected *p* values were calculated using the Benjamini-Hochberg method

| **Table S8. Significantly over-represented biological pathways and candidate genes identified in *Min*/D** | | | |
| --- | --- | --- | --- |
| **Pathway name** | **Database** | ***P* value (corrected)** | **Candidate genes** |
| Bile acid and bile salt metabolism | Reactome | 8.16 x 10^-2^ | *Cyp39a1, Fabp6* |
| Platelet aggregation | Reactome | 8.76 x 10^-2^ | *Rapgef4, Syk* |
| Integration of energy metabolism | Reactome | 9.28 x 10^-2^ | *Cacna1a, Cacna1c, Rapgef4* |

Candidate genes are genes that are found in the over-represented pathways and contained SNPs or indels private to the *Min*/D line.

Corrected *p* values were calculated using the Benjamini-Hochberg method.

| **Table S9. RT-qPCR primer/probe sets for mouse *Fabp6*, *Cyp39a1,* and *Hprt*** | | | |
| --- | --- | --- | --- |
| **Gene (IDT assay no.)** | **Probe** | **Primer 1** | **Primer 2** |
| *Fabp6* (Mm.PT.58.41459866) | 5’-/56-FAM/CTCCACCAA/ZEN/CTTGTCACCCACGA/3lABkFQ/-3’ | CCCAACTATCACCAGACTTCG | GCCAGCCTCTTGCTTACG |
| *Cyp39a1* (Mm.PT.58.28626812) | 5’-/56-FAM/TGCTATGGG/ZEN/AAACCGAATGACCTTTGT/3lABkFQ/-3’ | GATTGGAGCTGGACTTGAGTT | GAAGCACATTGATTCCTTCTTCT |
| *Hprt* (Mm.PT.39a.22214828) | 5’-/5HEX/CTTGCTGGT/ZEN/GAAAAGGACCTCTCGAA/3lABkFQ/-3’ | AACAAAGTCTGGCCTGTATCC | CCCCAAAATGGTTAAGGTTGC |
